# Sub‐Neuronal Network Profiling of Extracellular Vesicle Release Using a Compartmentalized Neurofluidic Platform

**DOI:** 10.1002/adbi.202500381

**Published:** 2026-02-18

**Authors:** Zeynep Malkoc, Esther Stopps, Prince M. K. Asamoah, Stephanie E. McCalla, Anja Kunze

**Affiliations:** ^1^ Department of Chemical and Biological Engineering Montana State University Bozeman Montana USA; ^2^ Department of Chemistry and Biochemistry Montana State University Bozeman Montana USA; ^3^ Department of Electrical and Computer Engineering Montana State University Bozeman Montana USA; ^4^ Montana Nanotechnology Facility Montana State University Bozeman Montana USA; ^5^ Optical Technology Center Montana State University Bozeman Montana USA

**Keywords:** Cryo‐EM, exosomes, extracellular vesicle profiling, neurofluidics, neuron‐derived micro‐RNA sequencing, okadaic acid

## Abstract

Extracellular vesicles (EVs) are membrane‐bound vesicles that are secreted by a wide range of organisms and cells, carrying cell‐specific receptors and molecular cargo such as proteins and nucleic acids. EVs have emerged as promising biomarkers for cancer and neurodegenerative disorders like Alzheimer's Disease (AD). Traditional methods for isolating neuron‐derived EVs from bodily fluids or conditioned media are based on bulk analysis methods, such as ultracentrifugation, isolation reagents, and immunoaffinity‐based techniques, and lack spatial resolution to capture localized secretion dynamics. Here, our neurofluidic platform compartmentalizes neuronal networks and enables spatially resolved analysis of EV profiling before subsequent traditional isolation and content screening. This intermediate resolution provides critical insights into localized sub‐neuronal EV secretion dynamics in cortical, hippocampal, and brainstem neurons. Using our platform, the influence of growth environment, cell maturation time, and exogenous stressors such as shear and biochemical stress can be unraveled. Biochemical stress is induced through okadaic acid (OA), a PP1A/PP2A inhibitor, which leads to hyperphosphorylation of proteins. In parallel, microRNA expression profiles are shown after OA treatment in primary neuron cultures, indicating an additional transcriptional response. These findings reveal regional differences in EV secretion dynamics associated with neuronal development and external stressors, including shear forces and PP1A/PP2A inhibition.

## Graphical abstract

This study employs a compartmentalized neurofluidic platform to map extracellular vesicle (EV) secretion dynamics with the aim of increasing the spatial resolution at sub‐neuronal locations. It examines how growth conditions, exogenous forces, and Okadaic acid treatment affect primary neurons' EV release. The study reveals spatially resolved EV dynamics and highlights potential miRNA biomarkers linked to tau‐related neurodegeneration.

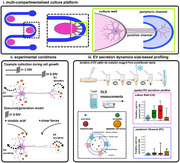

## 1 Introduction

Extracellular vesicles (EV) are lipid cargo carriers released by cells, which present a promising target for cell‐based diagnostics in fields such as cancer [1, 2, 3, 4], immunotherapy [5, 6, 7], and neurodegeneration [8, 9, 10, 11]. These vesicles are typically extracted from human blood serum, human cerebrospinal fluid, urine, or a conditioned cell culture medium to obtain EVs [12, 13, 14, 15, 16]. The resulting cluster of extracted EVs exhibits significant heterogeneity in terms of cell origin and size. Based on their physical appearance, EVs can be classified into four subgroups: exomeres (ranging from 15–25 nm in radius), exosomes (25–75 nm), microvesicles (50–500 nm), and apoptotic cell bodies (500–2500 nm) [17, 18]. Each of these sub‐groups of EVs plays a key role in mammalian cells, such as cell‐to‐cell communication [19, 20, 21, 22], cell content transfer (proteins, peptides, DNA, RNAs, or microRNAs) [6, 10, 23, 24], and cell apoptosis [25, 26]. In the brain, exosomes are known to be involved in neurogenesis [27], stress signaling [28], neurodegeneration [8, 29], and communication between neuronal and non‐neuronal cells [21]. Additionally, microvesicles have been associated with synaptic plasticity and neuroinflammation [30]. One of the most prevalent forms of neurodegeneration is Alzheimer's Disease (AD), which is characterized by the hyperphosphorylation of tau proteins and the accumulation of amyloid‐beta plaques. EVs have been found to carry tau proteins, amyloid‐beta (AB) peptides, and gene‐regulating micro‐RNA (miRNA) through the extracellular space, facilitating material transfer between neuronal cells [24, 31, 32, 33, 34].

With their versatile role in brain cell communication and signal transmission, EVs are uniquely suited to be utilized as biomarkers for neurodegenerative events. However, the heterogeneous nature of EVs poses challenges in tracing their specific subgroups or content to a defined neurodegenerative state. Furthermore, the progression of neurodegenerative disease is deeply entangled in a complex neuronal network [35]. A mature neuronal network consists of the neuronal bodies (soma), non‐neuronal supporter cells, and a dense network of neurites (axons and dendrites) that form synaptic connections. Typically, this neuronal network spans over millimeters in spatial dimensions. The release of EVs carrying neurodegenerative signals near the cell soma versus near the axonal tip can result in significant differences in cellular responses, leading to inflammation [30] or further degeneration [10, 35]. Therefore, capturing and profiling the heterogeneity of EVs released from different subnetwork locations is needed to advance EV‐based diagnostics for neurodegenerative diseases.

One of the common techniques to advance EV isolation is based on microfluidic systems. In microfluidic platforms, microchannels and microstructures can be utilized to filter, trap, selectively capture, or displace EVs based on their different sizes [17, 36, 37, 38, 39].

Subsequently, EVs can be further lysed for additional content analysis. So far, microfluidic techniques to capture EVs have been successfully applied to cancer diagnostics [18, 40] and personalized medicine [39]. However, microfluidic‐based EV profiling has been mainly demonstrated with pre‐processed, highly heterogeneous samples (cerebrospinal fluid, serum, or blood samples) rather than a focus on streamlined methodologies to investigate more precise spatial and temporal characterizations of EV secretion profiles. Selectively profiling the nature and content of EVs from subnetwork locations of complex matured neurite cultures has not been addressed.

Here, we present a microfluidic‐based assay, called the neurofluidic device, which utilizes a compartmentalized neurite culture to cultivate brain‐derived cells. Our platform allows for the capture and longitudinal profiles of EVs from specific subnetwork locations, offering a unique approach to study EV secretion dynamics in defined microenvironments. Growing neural cells in microchannels to compartmentalize their dendrites and somas from long‐reaching axons (>500 µm) has been a common method to organize the otherwise random formation of neuronal cell growth in vitro [41, 42, 43, 44, 45, 46]. By introducing biochemical factors or exogenous forces such as shear forces, we can elicit changes in neuronal behavior and investigate the response of the sub‐compartments to these perturbations. In this study, we used okadaic acid (OA), a known inhibitor of PPA1 and PP2A, to induce a biochemical stressor in a spatially controlled manner within the microfluidic environment. We hypothesized that exosomes and other extracellular vesicles would be secreted differently by subnetwork compartments, and the neurofluidic platform allows us to monitor EV secretion at a subcellular resolution [41, 42, 43, 47]. In a compartmentalized microfluidic platform, Kunze et al. have shown spatial inhibition of protein phosphatases PP1 and PP2A through OA in the distinct subnetwork neuronal compartments grown from cortical neurons, employing an Alzheimer‐like tauopathy model in primary neurons [42]. This phosphatase inhibition results in the spreading of hyperphosphorylated Tau proteins and the subsequent degeneration of the healthy/untreated network by preventing the removal of the phosphate groups that are added by kinases, leading to an accumulation of phosphorylated proteins and altered signaling [48, 49]. Rather than focusing on transport mechanisms of hyperphosphorylated Tau, we utilized OA as a tool to induce a controlled disruption of cellular signaling pathways, allowing us to investigate the subsequent effects on EV secretion rates in neurons. We isolated EVs from a conditioned culture media and mapped EV size distribution and secretion rates in both standard Petri dish cultures and our neurofluidic platform.

Additionally, we investigated the effects of different growth environments and biochemical stressors on EV secretion in these two growth environments using cortex, hippocampus, and brainstem primary neurons, exploring differences in selective vulnerability and the regional responses [50, 51, 52]. To further characterize the disruption of cellular pathways induced by OA, we measured neuronal miRNA profiles in cells under two distinct conditions: healthy neurons and 24 h after OA treatment. miRNAs are small (22‐nucleotide), non‐coding RNA molecules that are involved in gene regulation and signaling [52]. The dysregulation of miRNAs has been connected to many different disease states, such as cardiovascular diseases [50, 51], cancer [53, 54, 55], and neurodegenerative diseases, including AD [56, 57]. miRNAs are also relatively stable and present in many different body fluids, making them promising biomarkers for disease diagnosis and progression monitoring [58]. We were particularly interested in measuring miRNA dysregulation to further illuminate the neuronal responses to OA‐induced stress and inflammation and connect these responses to the EV secretion rates. This investigation offers a comprehensive characterization of miRNA expression under OA treatment. It can be used in further studies on miRNA content in EVs tied with their secretion patterns resulting from exogenous cellular modulations.

Overall, our study contributes to a gap in understanding neuronal EV secretion rates between simplified 2D traditional in vitro culture models and more biologically relevant, complex growth platforms. By profiling EV secretion rates and microRNA (miRNA) expressions across cortical, hippocampal, and brainstem neurons under both biochemical and mechanical stress, we aim to resolve region‐specific responses and spatially distinct EV secretion patterns. By providing a cost‐effective, reliable, and robust method for profiling EVs from maturing neuronal networks, this approach has the potential to advance the EV‐based biomarker space and optimize therapeutic strategies for neurodegenerative diseases and disorders.

## 2 Results and Discussion

### 2.1 The Secretion Rate of EVs Varies Across Brain Regions as Neurons Mature

**FIGURE 1:**
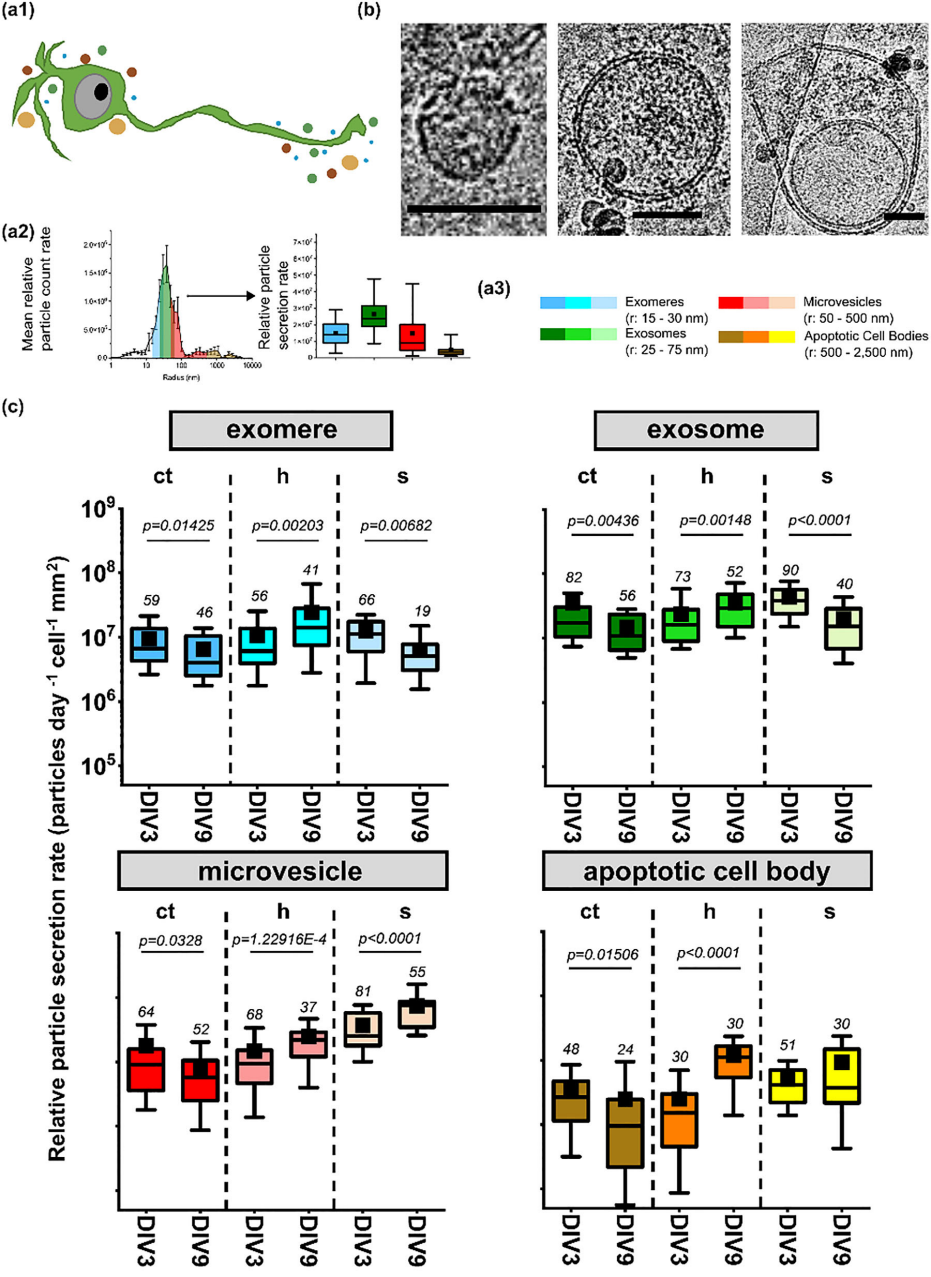
Relative secretion profile of extracellular vesicles (EVs) from distinct brain tissue regions cultured in Petri dishes. (a1) A schematic shows a single neuronal cell and size‐based characteristics of EV secretion. (a2) Size‐based EV profiling from conditioned media uses an EV extract and data post‐processing to yield raw intensity profiles (particle count rate) from Dynamic Light Scattering and size‐based secretion rate classification. (a3) Based on the hydrodynamic radius, EVs were characterized as exomeres (15–30 nm in radius), exosomes (25–75 nm), microvesicles (50–500 nm), and apoptotic cell bodies (500–2500 nm). (b) Representative cryo‐EM images showing different size‐based EV types. Scale bar = 50 nm. (c) Box plots show relative particle secretion rates from three distinct tissue culture regions (ct = cortex, h = hippocampus, s = brainstem) as neurons grew in Petri dishes (3 DIV, 9 DIV). Box plots whiskers are at 90^th^ and 10^th^ percentiles, n_biological sample_ = 3 – 10, N_measurements_  =  7. Significant p‐values (non‐parametric Mann‐Whitney U test, significance level at *α* = 0.05) and the number of data points are shown.

EV analysis often focuses on isolating nanometer‐sized small EVs (such as exosomes) from cell culture media, serum, bodily fluids, or cerebrospinal fluids (CSF) [11, 12, 13, 59, 60]. To separate exosomes, various protocols have been developed to optimize for higher purity and exosome yields, including ultracentrifugation [14, 61], immunoaffinity capture [62, 63], size exclusion chromatography [64], density gradient separation [65], or the use of commercial exosome isolation reagents [66]. However, these approaches require further analysis of the quantitative and qualitative characterization of other EV types presented in the samples. To assess different EVs present in conditioned brain cell culture media (Figure 1a1), we used a commercial exosome isolation reagent and analyzed the resulting isolated EVs using dynamic light scattering (DLS) to gain relative particle counts for each of the four size‐based EV classes (Figure 1a2,a3). We added a data post‐processing step to obtain a comprehensive EV profile based on the hydrodynamic radius of all vesicular, non‐vesicular components secreted by primary neurons to our post‐isolation DLS analysis. Within our sample sets, these extracellular nanoparticles (vesicular, non‐vesicular components) with a radius between 15 and 30 nm were referred as “*exomeres*”. Next, nanoparticles between 25 and 75 nm were named “*exosomes*”, between 50 and 500 nm “*microvesicles*”, and between 0.5 and 2.5 µm “*apoptotic cell bodies*”, respectively (Figure 1a3). Furthermore, to validate the sensitivity of the DLS instrument, we ran control experiments with microparticles and showed that DLS is quantitatively sensitive to particle concentration changes (Figure S1). We then validated the vesicle extraction efficiency of the commercial isolation kit by comparing EV isolation using the commercial reagent with isolation using EV‐free PBS in place of the commercial reagent (Figure S2). The DLS output with and without the exosome isolation reagent showed that this commercial method co‐isolated other EV classes, but selectively concentrated our classified exosomes and exomeres. It is worth noting that the centrifugation step at 2000 x g could result in loss of apoptotic bodies, but Figure S2 shows that some apoptotic bodies remain. In summary, this isolation and analysis process enabled us to determine the relative number of particles isolated in four selected EV classes over time: exomeres, exosomes, microvesicles, and apoptotic cell bodies.

**FIGURE 2:**
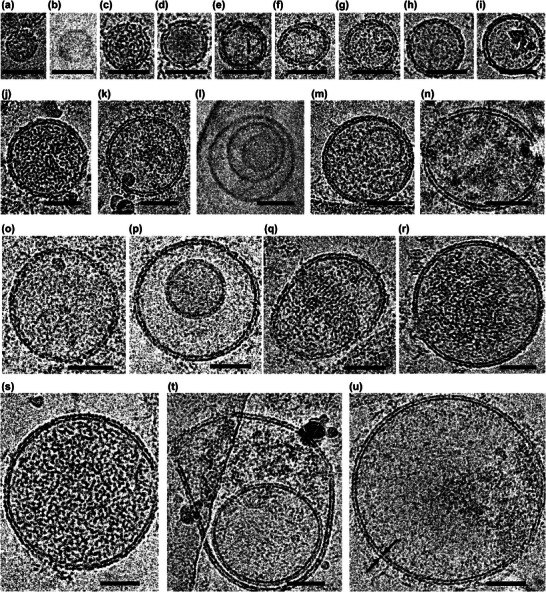
Representative cryo‐EM images of extracellular nanoparticles and vesicles. (a—u). Vesicles are arranged from smallest (a) to largest (u). Scale bar = 50 nm. Panels (a) and (b) indicate single leaflet vesicles, which were less than 50 nm in diameter, and showed a 3.5 nm leaflet thickness, potentially corresponding to exomeres. (c‐k,m‐o,q‐s and u) Cryo‐EM images show single lamellar vesicles. EV size distribution ranged from 33 nm to more than 244 nm (panel u) in diameter, with an average thickness of 5 nm between the inner and outer leaflets of the lipid bilayer (black arrows in panel u). (h,l,m,p, and t) Cryo‐EM images show multi‐lamellar vesicles.

Next, we imaged our extracted extracellular nanoparticle suspension with cryo‐EM to confirm variations in extracellular particle size (Figures 1b and 2). Throughout our study, the EV size profile represents the average relative particle count based on seven measurement repetitions with a minimum of three biological repetitions. EV relative particle counts are determined by summing the quantities within each size‐based class, normalized to the cell density and secretion time (per day). We subsequently compare these EV counts over time and across subcellular locations and extract secretion rates, including in experiments with serum supplementation in the culture media. This process flow provides an approximate method to assess EV secretion rates under cell stress, neurodegeneration, or enhanced cell activity.

To capture differences in EV secretion profiles based on culture days and brain cell types, we performed dissociated cultures from three distinct rat brain tissue regions. The cerebral cortex (ct), hippocampus (h), and brainstem (s) were selected for this study due to their well‐established involvement in AD‐related neurodegeneration and their differential vulnerability to its effects. To investigate how these regional differences influence EV secretion dynamics, we captured EV profiles from culture supernatant at 3 and 9 days in vitro (Figure 1c; Figure S3). The 3 DIV time point was chosen to represent an early stage of neuronal development, prior to the formation of mature networks. These early secretion dynamics were compared to those at 9 DIV, when neurons form denser and considerably more mature networks (Figure S4).

Analysis of normalized relative particle counts revealed distinct EV secretion profiles across cell types, as seen in Figure 1c. In the cortex, the rate of relative exosome secretion decreased as cells matured from 3 to 9 DIV, and this trend was repeated across all the other vesicle types. The relative exomere secretion rates in the cortex showed a 1.66 fold decrease (3 DIV: median = 6.69 × 10^6^, IQR = 9.26 × 10^6^, 9 DIV: median = 4.02 × 10^6^; IQR: 7.92 × 10^6^, whereas exosomes showed a 1.59 fold decrease (3 DIV: median = 1.72 × 10^7^, IQR = 2.01 × 10^7^; 9DIV: median = 1.08 × 10^7^, IQR = 1.69 × 10^7^). Microvesicle secretion rate showed a 1.57 fold decrease (3 DIV: median = 9.11 × 10^6^, IQR = 1.21 × 10^7^; 9 DIV: median = 5.77 × 10^6^, IQR = 8.01 × 10^6^). Similarly, apoptotic cell body secretion rate showed a reduction in the cortical neurons, with a 2.8 fold decrease (3 DIV: median = 2.67 × 10^6^, IQR = 3.57 × 10^6^; 9 DIV: median = 9.52 × 10^5^, IQR: 2.25 × 10^6^).

In contrast, hippocampal neurons exhibited a significant increase in the secretion rate of apoptotic cell bodies at 9 DIV, as high as 7.42 fold (3 DIV: median = 1.52 × 10^6^, IQR = 2.55 × 10^6^; 9 DIV: median = 1.13 × 10^7^, IQR = 1.11 × 10^7^). Furthermore, the secretion rates of exomeres, exosomes, and microvesicles were also elevated in the hippocampus at the later time point, 9 DIV. While the relative secretion rate of the exomeres showed a 2.3 fold increase (3 DIV: median = 6.08 × 10^6^, IQR = 9.65 × 10^6^; 9 DIV: median = 1.41 × 10^7^, IQR = 2.06 × 10^7^), exosome secretion rate increased about 1.82 fold in the hippocampus (3 DIV: median = 1.61 × 10^7^, IQR = 1.88 × 10^7^; 9 DIV: median = 2.92 × 10^7^, IQR = 3.31 × 10^7^). Lastly, the microvesicles exhibited an increase of 2.31 fold, similar to the exomeres (3DIV: median = 9.45 × 10^6^, IQR = 1.05 × 10^7^; 9 DIV: median = 2.19 × 10^7^, IQR = 1.7 × 10^7^). In the brainstem, the secretion rates of exosomes and exomeres showed a decrease, while microvesicle secretion increased at 9 DIV, and the apoptotic cell bodies remained relatively stable between 3 and 9 DIV. The decrease in the relative exomere secretion rate was recorded to be 2.21 fold (3 DIV: median = 1.13 × 10^7^, IQR = 1.15 × 10^7^; 9 DIV: median = 5.11 × 10^6^, IQR = 4.66 × 10^6^), and 2.5 fold for the exosomes in the brainstem (3DIV: median = 3.83 × 10^7^, IQR = 3.31 × 10^7^, 9 DIV: median = 1.53 × 10^7^, IQR = 2.19 × 10^7^). With the microvesicles, the increase in the secretion rate was recorded as a 3 fold increase (3 DIV: median = 2.53 × 10^7^, IQR = 3.97 × 10^7^; 9 DIV: median = 7.63 × 10^7^, IQR = 5.19 × 10^7^).

Overall, cortical neurons showed a decreased secretion rate of all EV subtypes as they got closer to a mature state. However, the hippocampal neurons showed significant increases in EV secretion rates, especially in the apoptotic bodies (7.4 fold increase). Brainstem neurons showed a mixed trend with decreased secretion of exosomes and exomeres and increased microvesicle release over time. These results may suggest that there is a brain region‐specific maturation response in the EV dynamics in primary rat neurons.

### 2.2 Qualitative Profiling of EVs From Brain Cell Conditioned Medium by Cryo‐electron Microscopy (Cryo‐EM)

As a qualitative analysis, samples were vitrified and imaged by cryo‐EM. The resulting micrographs revealed a broad size distribution of extracellular vesicles (Figure 2a–u). The most abundant population ranged from approximately 50 to 150 nm in diameter, consistent with the size range usually associated with exosomes. A small number of vesicles measuring 30–50 nm were also observed, which may correspond to exomeres. Additionally, a secondary population of larger vesicles greater than 150 nm in diameter was identified, which likely represents microvesicles, given their characteristic size range [17, 18, 19]. The lipid bilayer membranes of the vesicles exhibited an average thickness of approximately 5 nm (Figure 2u), consistent with canonical bilayer structures. The majority of EVs were single lamellar vesicles; however, a number of multi‐lamellar vesicles were also observed (Figure 2h,l,m,p,t). Some of the vesicles (Figure 2m,q,r,s) appear to contain electron‐dense material as indicated by the dark matrix observed in their lumen. Overall, the cryo‐EM survey revealed vesicles whose size distribution was consistent with 4 exomeres (4 %), 53 exosomes (58 %), and 35 microvesicles (38 %) from an EV secretion sample from the cerebral cortex.

### 2.3 Okadaic Acid (OA) Treatment Stimulates an Earlier Response in EV Secretion in the Hippocampus

**FIGURE 3:**
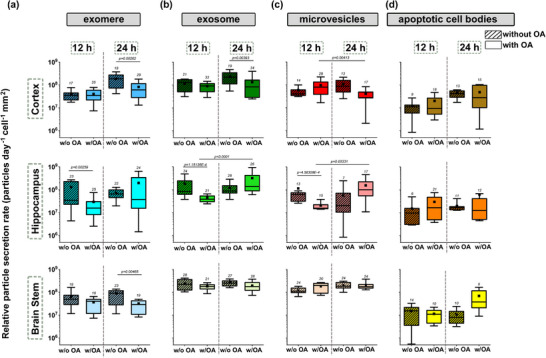
Impact of okadaic acid (OA) treatment on extracellular vesicle secretion dynamics in neuronal cultures from three different brain tissue regions (cortex, hippocampus, and brainstem). Boxplots show relative particle secretion rates in the four classified EV categories (a) exomeres, (b) exosomes, (c) microvesicles, (d) apoptotic cell bodies post 12 and 24 h 100 nM OA treatment, without (w/o = sham), and with (w/). Sham manipulations (12 and 24 h) served as a control to assess the effect of mechanical stress on EV secretion. For each boxplot, n_biological sample_ ≥ 3, N_measurements_ = 7. Statistical significance (α = 0.01) was determined using a non‐parametric Mann‐Whitney U test, and an unpaired t‐test for the normally distributed data sets (Shapiro‐Wilk normality test).

Differential vulnerability in neurodegeneration is determined by regional differences of neurodegenerative hallmarks in distinct brain tissues [67]. This vulnerability is specifically associated with tauopathies, where tau hyperphosphorylation leads to the degeneration of the neuronal networks [68, 69]. Furthermore, it is known that EVs play a role in the progression of tauopathies. Therefore, we focus on examining EV profiles from cell cultures derived from distinct brain tissue regions (cortical, hippocampal, and brainstem regions) at 9 days in vitro (DIV) with the addition of a biochemical reagent, okadaic acid (OA). One characteristic feature of in vitro OA‐induced PP2A inhibition is the rapid degeneration of the neurite networks. We observed neurite network degeneration through the disintegration of neurite branches, leaving dotted puncta, especially in cortical and hippocampal neuronal cell cultures, based on brightfield images taken at 9 DIV pre‐treatment and 24 h post‐OA incubation (Figure S5, OA: 100 nm, 60 min). We then collected conditioned cell culture media samples 12 and 24 h after the OA treatment, and normalized relative particle secretion rates to the cell density and the daily secretion (Figure 3a–d). This experimental procedure involves frequent pipetting, which can result in shear stress on the cells. Therefore, we compared a sham group to a non‐treated Petri dish sample from 9 DIV to be able to test whether the cells were reacting to the shear forces caused by frequent pipetting (Figure S6). We found that vigorous pipetting can increase the rate of EV secretion for most EVs across all cell cultures from the three distinct brain tissue regions.

As shown in Figure 3, the OA treatment post 24 h yielded a significant decrease in exomeres particle secretion when compared to the sham condition in the cortex cultures (3 fold decrease, 24 h w/o OA: median = 1.87 × 10^8^, IQR = 1.99 × 10^8^; 24 h w/OA: median = 6.12 × 10^7^, IQR = 8.7 × 10^7^) and brainstem (3.12 fold decrease, 24 h w/o OA: median = 9.08 × 10^7^, IQR = 8.85 × 10^7^; 24 h w/OA: median = 2.91 × 10^7^, IQR = 3.15 × 10^7^) (Figure 3a). However, in the hippocampus, the decrease in exomere secretion rate was observed earlier, at the 12 h time point (2.18 fold decrease, 12 h w/OA: median = 3.46 × 10^7^, IQR = 2.05 × 10^8^; 12 h w/OA: median = 1.58 × 10^7^, IQR = 2.22 × 10^7^, Figure 3a).

For the exosomes, in the cortex, we see a later response to OA treatment at the 24 h with a 2.66 fold decrease (24 h w/o OA: median = 2.2 × 10^8^, IQR = 2.32 × 10^8^; 24 h w/OA: median = 8.27 × 10^7^, IQR = 1.36 × 10^8^), and (Figure 3b), we see that the hippocampus reacts to the treatment earlier at 12 h (2.2 fold decrease, 12 h w/o OA: median = 8.48 × 10^7^, IQR = 1.82 × 10^8^; 12 h w/OA: median = 3.87 × 10^7^, IQR = 2.35 × 10^7^, Figure 3b). No significant changes in exosome secretion rates in the brainstem were noted (Figure 3b). We have also recorded a significant 3.58 fold increase in exosomes between the 12 h and 24 h OA treatment groups (24 h w/OA: median = 1.38 × 10^8^, IQR = 3.37 × 10^8^) in the hippocampus (Figure 3b). This indicates that even though there was initially suppressed secretion compared to the baseline, in the hippocampus, the exosome secretion recovers and even increases transiently.

In the cortex, the microvesicle secretion rate elevated slightly but did not show a significance compared to the sham control, however, our results showed a 2.87 fold inhibition in secretion rate between the OA treatment groups from 12 h treatment to 24 h treatment (12 h w/OA: median = 7.91 × 10^7^, IQR = 9.51 × 10^7^; 24 h w/OA: median = 2.76 × 10^7^, IQR = 2.17 × 10^7^) (Figure 3c). In the hippocampus, we observed an early time point response at 12 h resembling the exomere and exosome secretion rate profiles when compared to the sham. Once again, like the exomere and exosome secretion dynamics, the OA resulted in an early inhibition in microvesicle secretion rates (3.9 fold decrease, 12 h w/o OA: median = 6.21 × 10^7^, IQR = 4.31 × 10^7^; 12 h w/OA median = 1.59 × 10^7^, IQR = 7.42 × 10^6^). Additionally, in the hippocampus, we recorded a recovery in secretion rates for microvesicles, just like the exosomes (6.36 fold increase, 24 h w/OA: median = 1.01 × 10^8^_,_ IQR = 1.7 × 10^8^) (Figure 3c). There were no significant changes recorded in microvesicle secretion in the brainstem (Figure 3c).

Looking at the apoptotic cell bodies, we recorded no significant changes in secretion rates in the cortex, hippocampus, and brainstem. Compared to the other vesicle types, we could also note that apoptotic cell bodies were secreted less in the cortex and hippocampus (Figure 3d).

This experimental design allowed us to investigate EV release dynamics within a randomized neuronal growth environment. Our findings reveal distinct EV release profiles across cortical, hippocampal, and brainstem cultures following treatment with a biochemical agent, OA. Specifically, hippocampal cultures exhibited higher mean secretion rates of small EVs (exomeres and exosomes) at 24 h compared to cortical and brainstem cultures after OA treatment. The overall inhibition in EV secretion rates, especially of small EVs, may suggest either an increase in cellular uptake of EVs amongst the cells at the 12 and 24 h time points or a “silencing” response after OA treatment. Notably, the addition of OA did not significantly elevate the apoptotic cell body secretion rates overall. While these findings provide valuable insights into EV dynamics in a simplified 2D in vitro system, it is important to acknowledge the limitations of this model. As the next step in this study, we replicated the same experimental protocols within a compartmentalized and more organized growth environment, using our neurofluidic technology, where we achieve a higher density of cells. This allowed us to investigate whether these secretion dynamics reveal spatially specific and distinct patterns of EV secretion in primary neurons.

### 2.4 Spatial Compartmentalization of Neuronal Growth Reveals Distinct Local EV Secretion Profiles

**FIGURE 4:**
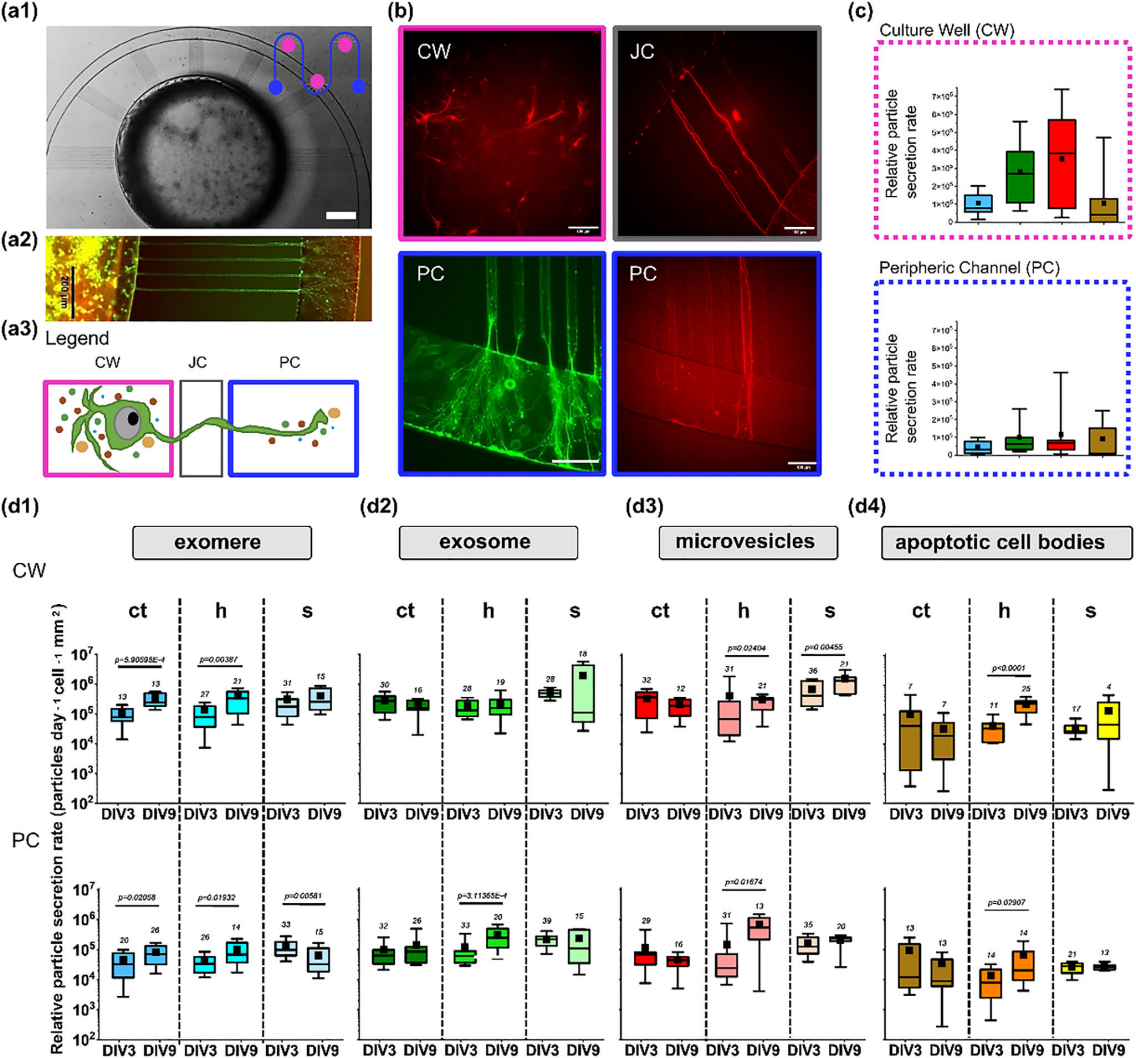
Boxplots depict the relative secretion rates of extracellular vesicles (EVs) in neuronal cultures grown in neurofluidic devices at 3 and 9 days in vitro (DIV). (a1) Phase contrast image shows neurofluidic design with one out of three culture wells (CW), junction channels (JC), and one peripheric channel (PC) compartments. Scale bar = 500 µm. (a2) False‐color fluorescence images showing neuronal cell bodies in the CW, neurite structures in the JC, and PC. Scale bar = 200 µm. (a3) Schematic of legend, not to scale. (b) Zoom‐in view of false‐color fluorescence images in CW, JC, and PC, (green: Fluo4‐AM, red: lipid staining). Scale bar = 100 µm. (c) Relative EV particle secretion rates from the three CW and the PC. The study included at least 3 neuronal cultures (n_biological_), and 7 measurements per sample (N_measurement_). Similar to neuronal cultures in Petri dishes, the cell media in the neurofluidic devices were replaced every 3 days (at 3 DIV, 6 DIV, and 9 DIV) after collecting samples from the culture well (CW), and peripheral channel (PC) compartments. The study focused on analyzing the spatial and temporal secretion patterns of four types of size‐based classified EVs: (a) exomeres, (b) exosomes, (c) microvesicles, and (d) apoptotic cell bodies from cell cultures from three different brain tissue reagions cortex (ct), hippocampus (h), and brainstem (s). A non‐parametric Mann‐Whitney U test was conducted using a significance level of α = 0.05.

To investigate whether different subcellular culture regions impact EV profiles, we seeded dissociated cell bodies from cortical, hippocampal, and brainstem tissues into the somatic well of an in‐house‐designed neurofluidic device (Figure 4a1–a3). Similar to what was seen in the literature [41, 42, 44, 45, 46, 70, 71, 72], neurite extensions from neuronal cells grew into the peripheral channel through a parallel channel system (Figure 4a2,4b). This platform allowed us to collect live‐cell, non‐dendritic specific samples independent from the somatic well for further EV isolation and profiling (Figure 4c,d1–d4).

We extracted relative particle counts from the live‐cell EV profiles, normalized by the cell density and the secretion rates for each of the four EV categories. As shown in Figure 4d1, in the cortex between 3 DIV and 9 DIV, there was a significant 3.1 fold increase in exomere secretion within the culture wells (3 DIV: median = 7.86 × 10^4^, IQR = 9.15 × 10^4^; 9 DIV: median = 2.45 × 10^5^, IQR = 3.15 × 10^5^). However, in the cortex, no significant change in the secretion rate was observed for other EVs as the cells matured (Figure 4d2–d4). This pattern repeated in the peripheric channel for the cortex, where we see a significant 2.1 fold increase in the exomere secretion (3 DIV: median = 3.33 × 10^4^, IQR = 6.47 × 10^4^; 9 DIV: median = 7.02 × 10^4^, IQR = 9.98 × 10^4^) (Figure 4d2–d4). In summary, the maturation of the cortex in dense networks did not significantly affect the secretion rate of all subtypes of EVs, except for the exosomes. This contrasts with the data in the Petri dish, which showed a small decrease in EV secretion rate during maturation.

As hippocampal neurons undergo maturation, an increase in the secretion of exomeres (4.21 fold increase, 3 DIV: median = 7.94 × 10^4^, IQR = 1.46 × 10^5^; 9 DIV: median = 3.34 × 10^5^, IQR = 4.57 × 10^5^), microvesicles (4.41 fold increase, 3 DIV: median = 7.21 × 10^4^, IQR = 2.6 × 10^5^; 9 DIV: median = 3.18 × 10^5^, IQR = 2.22 × 10^5^), and apoptotic cell bodies (6.94 fold increase, 3 DIV: median = 3.39 × 10^4^, IQR = 3.9 × 10^4^; 9 DIV: median = 2.35 × 10^5^, 1.68 × 10^5^) is observed in the culture wells (Figure 4d1–d4). In the peripheric channel, all the EV subtypes: exomere (2 fold increase, 3 DIV: median = 3.37 × 10^4^, IQR = 4.05 × 10^4^; 9 DIV: median = 6.81 × 10^4^, IQR = 1.35 × 10^5^), exosome (3.98 fold increase, 3 DIV: median = 6.16 × 10^4^, IQR = 5.38 × 10^4^; 9 DIV: median = 2.45 × 10^5^, IQR = 3.84 × 10^5^), microvesicle (2.25 fold increase, 3 DIV: median = 2.42 × 10^4^, IQR = 5.82 × 10^4^; 9 DIV: median = 5.44 × 10^5^, IQR = 9.26 × 10^5^), and apoptotic cell body (2.55 fold increase, 3 DIV: median = 8 × 10^3^, IQR = 1.92 × 10^4^; 9 DIV: median = 2.04 × 10^4^, IQR = 7.61 × 10^4^) secretion rate were found to increase (Figure 4d1–d4). Similar to the findings from the Petri dish data, hippocampal cells appeared to be potentially susceptible to apoptosis or higher particle shedding. Consistently, EV secretion by hippocampal cells increased in the peripheric compartment from 3 DIV to 9 DIV.

Finally, for the brainstem, in culture wells, secretion rates for exomeres, exosomes, and apoptotic cell bodies in the culture well showed no significance between 3 DIV and 9 DIV (Figure 4d1–d2,d4). The only increase in secretion rate was noted in microvesicles with a 3.19 fold increase in the culture well (3 DIV: median = 4.41 × 10^5^, IQR = 1.17 × 10^6^; 9 DIV: median = 1.41 × 10^6^, IQR = 1.35 × 10^6^, Figure 4d3). In the peripheric channel, the only significant change was recorded in the exomere secretion with a 2.97 fold decrease from 3 DIV (median = 9.81 × 10^4^, IQR = 1.17 × 10^5^) to 9 DIV (median = 3.30 × 10^4^, IQR = 9.29 × 10^4^, Figure 4d1). Exomere secretion rates in the Petri dish declined as cells matured in the brainstem (Figure 1c), and a similar pattern was observed in the peripheric channel (Figure 4d1). The increase in microvesicle secretion rates in the Petri dishes (Figure 1c) was reflected in the culture well for brainstem neurons within the neurofluidic device (Figure 4d3).

### 2.5 Mechanical Forces Alter EV Secretion Rates in the Neurofluidic Platform

**FIGURE 5:**
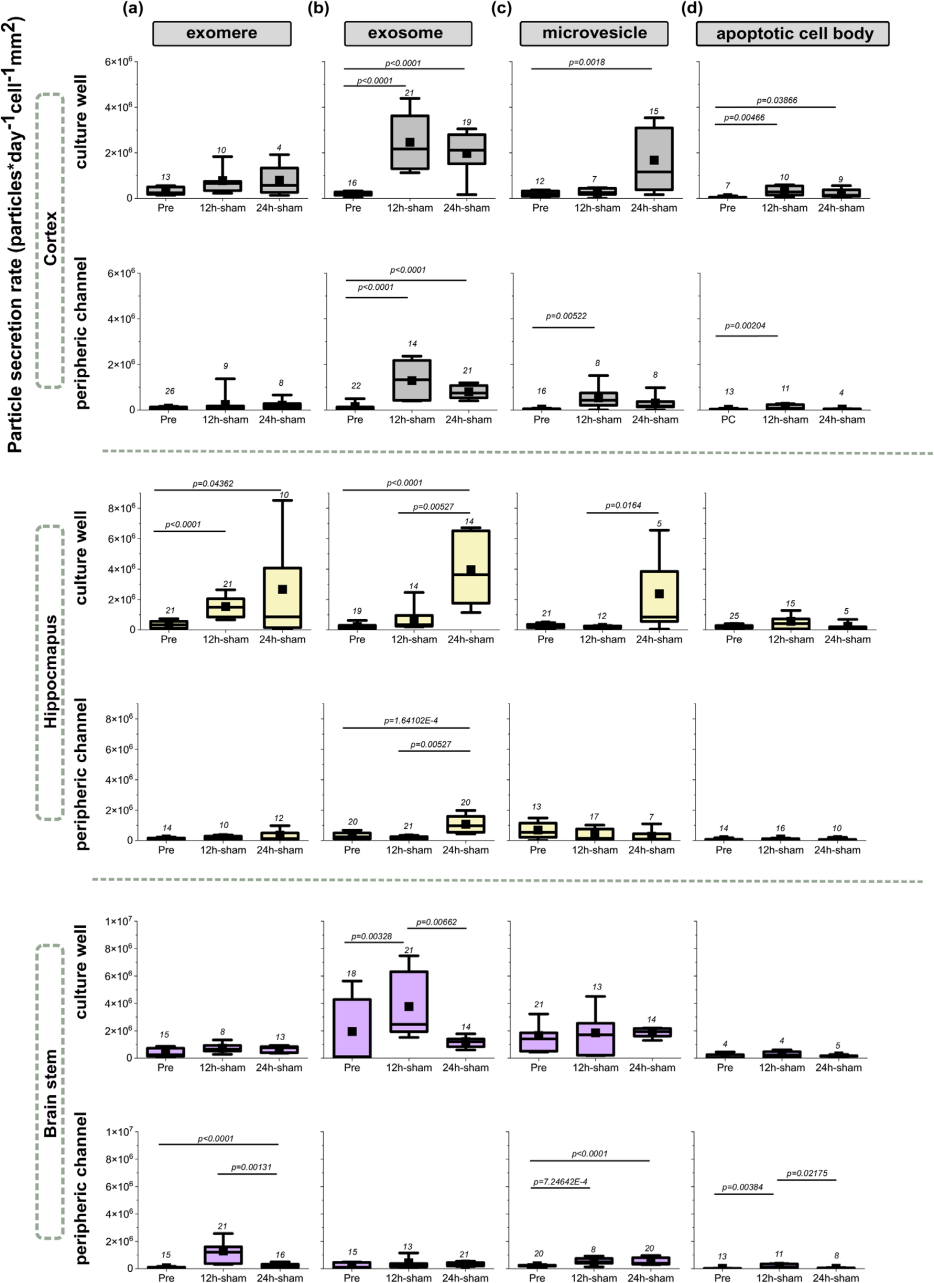
EV particle secretion rates under fluidic handling in the neurofluidic device. EV subtype profiles are shown based on size classification as (a) exomeres, (b) exosomes, (c) microvesicles and (d) apoptotic cell bodies, collected from the peripheric channel and culture well before any experimental fluidic handling (pre, 9 DIV), 12 h post sham condition (12h‐sham, fluidic handling, 1 h incubation with fresh culture media, w/o OA), and 24 h post sham condition (24h‐sham, fluidic handling, w/o OA). EVs were extracted from brain tissue region‐specific cell cultures: (a1‐d2) cortex, (a3‐d4) hippocampus, and (a5‐d6) brainstem at 9 DIV. Non‐parametric Kruskal‐Wallis ANOVA test was conducted at significance level α = 0.05.

Similar to the Petri dishes, we ran the same control experiments to account for the effect of mechanical forces caused by frequent pipetting while performing the OA‐treatment experiments in our neurofluidic platform. There are forces such as fluid shear forces and capillary forces applied to the cells in the somatic compartment, and the synaptic tips that are sitting at the peripheric channel during collecting samples. To account for the effect of these forces on neurons, we compared the “pre” baseline, where the cells do not get any treatment or go through frequent pipetting and media changes, to the “sham” group, where we employ the OA treatment experimental protocol without the addition of the reagent (Figure 5a–d).

In the cortex, both the culture well and the peripheric channel secretion were affected by the shear forces, especially for exosomes, microvesicles, and apoptotic cell bodies. In the cortical culture wells, exosome secretion at 12 h increased by 13.5 fold, and further increased at 24 h by 13.2 fold (Pre: median = 1.6 × 10^5^, IQR = 1.45 × 10^5^; 12 h sham: median = 2.17 × 10^6^, IQR = 2.32 × 10^6^; 24 h sham: median = 2.12 × 10^6^, IQR = 1.28 × 10^6^) compared to the pre‐baseline (Figure 5b1). In the peripheric, a similar pattern was seen with a 19.8 fold increase at 12 h, and an 11 fold increase at 24 h (pre: median = 4.71 × 10^4^, IQR = 9.64 × 10^4^; 12 h sham: median = 1.33 × 10^6^; IQR = 1.75 × 10^6^; 24 h sham: median = 7.44 × 10^5^, IQR = 5.41 × 10^5^) (Figure 5b2). Shear forces caused an increase in microvesicle secretion at the later time point at 24 h in the culture wells by a 5.82 fold (pre: median = 2 × 10^5^, IQR = 2.32 × 10^5^; 24 h sham: median = 1.16 × 10^5^, IQR = 2.71 × 10^6^) (Figure 5c1), while this increase was observed at 12 h in the peripheric channel by a 9.78 fold increase. (pre: median = 4.43 × 10^4^, IQR = 2.87 × 10^4^; 12 h sham: median = 4.33 × 10^5^, IQR = 5.39 × 10^5^) (Figure 5c2). The apoptotic cell body secretion rate in the culture well increased by a 14.64 fold at 12 h, and 7.31 fold at 24 h (pre: median = 1.92 × 10^4^, IQR = 4.99 × 10^4^, 12 h sham: median = 2.82 × 10^5^, IQR = 4.02 × 10^5^; 24 h sham: median = 1.41 × 10^5^, IQR: 2.83 × 10^5^) (Figure 5d1). This increase was only recorded at 12 h with an 8.3 fold increase in the peripheric channel (pre: median = 8.95 × 10^3^, IQR: 4.13 × 10^4^; 12 h sham: median = 7.43 × 10^4^, IQR = 1.83 × 10^5^) (Figure 5d2).

The hippocampal neurons demonstrated a more moderate response at the peripheral channel to mechanical stress. In the culture well, exomere secretion rate was affected by the mechanical forces with a 4.48 fold increase at 12 h, and a 2.58 fold increase at 24 h (pre: median = 3.34 × 10^5^, IQR = 4.57 × 10^5^; 12 h sham: median = 1.5 × 10^6^, IQR = 1.2 × 10^6^; 24 h sham: median = 8.62 × 10^5^, IQR = 3.92 × 10^6^) (Figure 5a3). Exosome secretion increased at 24 h by 23.2 fold, and we noted a significant 10.6 fold increase between 12 and 24 h sham samples (pre: median = 1.56 × 10^5^, IQR = 2 × 10^5^; 12 h sham: median = 3.43 × 10^5^, IQR = 7.03 × 10^5^; 24 h sham: median = 3.63 × 10^6^, IQR = 4.76 × 10^5^) (Figure 5b3). The same trend was seen in the peripheric channel with a 4 fold increase at 24 h, and a 7.8 fold increase between 12 and 24 h sham samples (pre: median = 2.45 × 10^5^, IQR = 3.84 × 10^5^; 12 h sham: median = 1.25 × 10^5^, IQR = 1.63 × 10^5^; 24 h sham: median = 9.78 × 10^5^, IQR = 1.05 × 10^6^) (Figure 5b4). Only significant change in microvesicle was recorded at the culture well between 12 and 24 h by a 5.75 fold increase in the secretion rate (12 h sham: median = 1.47 × 10^5^, IQR = 1.48 × 10^5^, 24 h sham: median = 8.46 × 10^5^, IQR = 3.29 × 10^6^) (Figure 5c3).

In the brainstem, effects of mechanical forces were more pronounced in the peripheric channel than in the culture well (Figure 5a5–d6). Exosome secretion in the culture well increased notably by 22.34 fold at 12 h, but subsequently decreased between 12 and 24 h by 2 fold (pre: median = 1.1 × 10^5^, IQR = 4.23 × 10^6^; 12 h sham: median = 2.47 × 10^6^, IQR = 4.38 × 10^6^; 24 h sham: median = 1.19 × 10^6^, IQR = 5.71 × 10^5^) (Figure 5b5). In the peripheric channel, frequent pipetting caused a 4.39 fold increase in exomeres secretion at 24 h, and we observed a significant decrease in exomeres secretion between 12 h and 24 h by 8.28 fold (pre: median = 3.03 × 10^4^, IQR = 9.29 × 10^4^; 12 h sham: median = 1.2 × 10^6^, IQR = 1.23 × 10^6^; 24 h sham: median = 1.45 × 10^5^, IQR = 2.33 × 10^5^) (Figure 5a6). Microvesicle secretion rates were increased by 2.16 fold at 12 h, and 1.7 fold at 24 h compared to the pre‐baseline in peripheric channel (pre: median = 2.33 × 10^5^, IQR = 6.71 × 10^4^, 12 h sham: median = 5.05 × 10^5^, IQR = 3.49 × 10^5^; 24 h sham: median = 3.97 × 10^5^, IQR = 4.72 × 10^5^) (Figure 5c6). Apoptotic cell body secretion increased at 12 h by 4.7 fold, and decreased between 12 and 24 h by 4.57 fold (pre: median = 2.58 × 10^4^, IQR = 8.6 × 10^3^; 12 h sham: median = 1.21 × 10^5^, IQR = 3.03 × 10^5^; 24 h sham: median = 2.66 × 10^4^, IQR = 3.98 × 10^4^) (Figure 5d6).

These results show that frequent mechanical stress can significantly alter EV secretion dynamics with region, sub‐neuronal location, and EV‐type specific sensitivity. Our results demonstrate that the cortical neurons appeared more responsive to mechanical stresses across multiple EV subtypes in both axonal and somatic locations. Meanwhile, hippocampal neurons exhibited a muted response at the axonal (peripheric) compartment, while the brainstem particularly had a more definite response at that location. In summary, these findings highlight the importance of controlling for exogenous mechanical stress artifacts when interpreting the EV secretion dynamics in vitro.

### 2.6 Subcellular Neuronal Compartments Show Differential EV Secretion Profiles After Okadaic Acid Treatment

**FIGURE 6:**
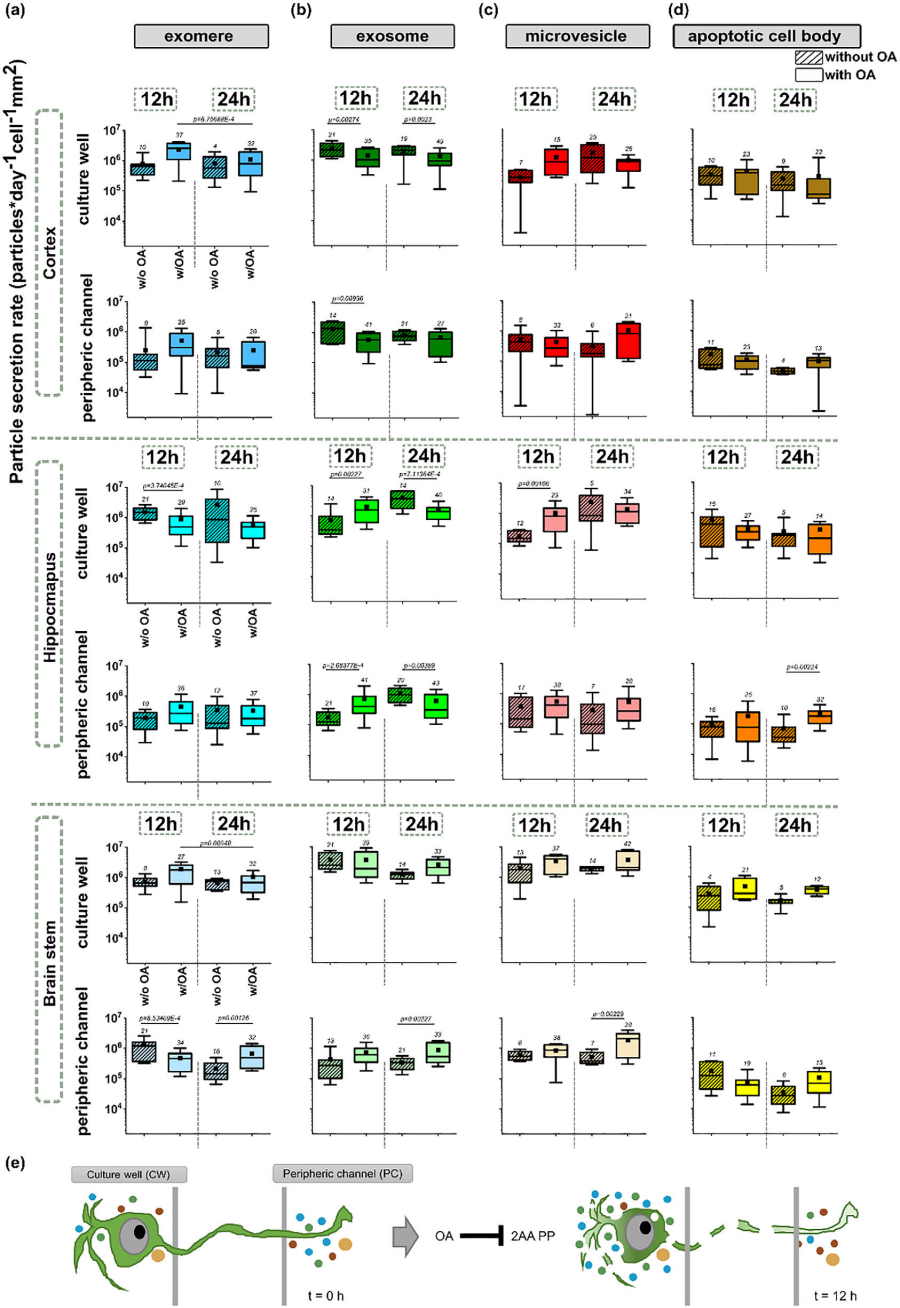
Extracellular vesicles (EV) secretion rates show distinct profiles for different brain tissue regions (cortex, hippocampus, brainstem), after 12 and 24 h following the okadaic acid (OA, protein phosphatase PP2A inhibition) treatment in the culture well in the neurofluidic devices. OA‐treated cells are denoted w/OA, and healthy cells that experienced a sham treatment with unconditioned media only are denoted w/o OA. We show the spatial‐temporal profiles for four distinct types of EVs: (a) exomeres, (b) exosomes, (c) microvesicles, and (d) apoptotic cell bodies. A non‐parametric Mann‐Whitney U‐test was conducted at an adjusted significance level (Bonferroni correction) of *α = 0.01* due to the number of comparisons made. (e) Schematic of a potential mechanism for OA‐induced differential EV section profile fingerprint across distinct neuronal cell body compartments.

Several studies have demonstrated that okadaic acid (OA) can effectively induce tauopathy resembling Alzheimer's disease in both rodent and human cell cultures and tissues [42, 48, 49, 73, 74]. These induced tauopathies can be modeled through the inhibition of phosphatase activity by OA [42, 49, 75, 76]. While the effects of OA on overall cellular function have been studied and differential miRNA transcription was shown, it remains unknown if OA treatment would induce differential subcellular EV secretion profiles. To investigate this question, we seeded primary cortical, hippocampal, and brainstem cells in the somatic well in the neurofluidic device and exposed the cell bodies in the culture well to OA (100 nM, 60 min) on 9 DIV. Samples from the conditioned media were collected from the sham (w/o OA) and after treatment (w/OA) at 12 and 24 h from the somatic (culture well: CW) and peripheral compartments (PC) of the neurofluidic devices (Figure 6a–d).

In the neurofluidic devices, there were no significant changes recorded in exomere secretion in the cortex in both the culture well and peripheric channel compared to the sham baseline (Figure 6a). However, the treatment group showed a 3.24 fold decrease in the culture well in the cortex (Figure 6a, 12 h w/OA: median = 2.54 × 10^6^, IQR = 2.58 × 10^6^; 24 h w/OA: median = 7.84 × 10^5^, IQR = 1.59 × 10^6^). This might suggest OA treatment may cause a temporal shift in exosome secretion in the somatic compartment where the cortical neurons are densely seeded. In the hippocampus, exomere secretion was inhibited by 3.04 fold compared to the sham baseline (w/o OA) at the earlier point of 12 h (w/o OA: median = 1.5 × 10^6^, IQR = 1.2 × 10^6^; w/OA: median = 4.93 × 10^5^, IQR = 8.48 × 10^5^, Figure 6a), and there were no significant changes recorded in the culture well and peripheric channel except the early inhibitory effect in the culture well (Figure 6a). Lastly, in the brainstem, an inhibitory effect on exomere secretion rate was observed between the treatment groups in the culture well. In the peripheric channel, an additional inhibition of exomere secretion was seen at 12 h, followed by an increase in secretion rate at 24 h when compared to the baseline sham groups. No significant effect of the OA treatment was found in the peripheral channel (Figure 6a).

The exosome secretion rate significantly decreased in the cortex culture well for the treatment group at both 12 and 24 h compared to the sham group without OA treatment; with a 2.1 fold decrease at 12 h (w/o OA: median = 2.17 × 10^6^, IQR = 2.32 × 10^6^; w/OA: median = 1.03 × 10^6^, IQR = 1.77 × 10^6^), and a 2.26 fold decrease at 24 h (w/o OA: median = 2.12 × 10^6^, IQR = 1.25 × 10^6^, w/OA: median = 9.73 × 10^5^, IQR = 1.02 × 10^6^, Figure 6b). The same inhibitory effect was observed at 12 h in the peripheric channel with a 2.33 fold decrease (w/o OA: median = 1.33 × 10^6^, IQR = 1.75 × 10^6^; w/OA: median = 5.73 × 10^5^, IQR = 7.14 × 10^5^, Figure 6b). In the hippocampus, we observed an initial 4.55 fold increase in the secretion rate of exosomes after OA treatment at the early point of 12 h (w/o OA: median = 3.43 × 10^5^, IQR = 7.03 × 10^5^; w/OA: median = 1.56 × 10^6^, IQR = 1.12 × 10^6^), and this was followed by a 2.6 fold decrease in secretion rate at 24 h in the culture well (w/o OA: median = 3.63 × 10^6^, IQR = 4.76 × 10^6^; w/OA: median = 1.4 × 10^6^, IQR = 1.12 × 10^6^, Figure 6b). This trend was repeated for the exosome secretion in the hippocampus at the peripheric channel with a 3.21 fold increase at 12 h (w/o OA: median = 1.25 × 10^5^, IQR = 1.63 × 10^5^; w/OA: median = 4.02 × 10^5^, IQR = 6.26 × 10^5^), and 3.15 fold decrease at 24 h (w/o OA: median = 9.78 × 10^5^, IQR = 1.05 × 10^6^, w/OA: median = 3.11 × 10^5^, IQR = 8.31 × 10^5^, Figure 6b). In the brainstem, there were no significant changes in exosome secretion rate in the culture well, whereas there was an increase in exosome secretion rate at 24 h compared to the sham group in the peripheral compartment (Figure 6b).

For the microvesicles, there were no significant changes observed in the cortex (Figure 6c). Although it was not noted to be significant, the microvesicle secretion pattern in culture wells showed a similar pattern to the cortex in Petri dishes. In the hippocampus, the only noted change in the microvesicle secretion rate was a 5.48 fold increase in the culture well at the early time point, 12 h after the OA treatment (w/o OA: median = 1.47 × 10^5^, IQR = 1.48 × 10^5^; w/OA: median = 8.06 × 10^5^, IQR = 1.23 × 10^6^, Figure 6c). No significant changes were observed in the peripheric channel for the hippocampal cells (Figure 6c). An increase in microvesicle secretion was also observed 24 h after OA treatment in the peripheric channel in the brainstem (w/o OA: median = 3.97 × 10^5^, IQR = 4.72 × 10^5^; w/OA: median = 2.09 × 10^6^, IQR = 2.56 × 10^5^, Figure 6c), whereas no significant changes were seen in the culture well for the brainstem (Figure 6c).

While in microfluidics, apoptotic cell body secretion was not affected by the OA treatment in the cortex, brainstem, and the culture well in the hippocampal cells, we recorded a 5 fold increase in the secretion rates of apoptotic bodies in the peripheric channel at 24 h after treatment in the hippocampus (w/o OA: median = 3.6 × 10^4^, IQR = 5.3 × 10^4^; w/OA: median = 1.82 × 10^5^, IQR = 1.6 × 10^5^, Figure 6d).

Overall, our findings showed that the brain tissue cultures in the neurofluidic devices stimulate different relative EV secretion profiles than in Petri dish cultures with and without the okadaic acid treatment (Figure S7). While the majority of EV secretion rate changes were seen in the culture wells for the cortex and hippocampus cell cultures, in the brainstem cultures, this spatial alteration showed itself in the peripheric compartment. This might suggest that, in the brainstem, axonal tips may be more sensitive to biochemical stress and PPA1/PP2A inhibition. With our neurofluidic device, we can tell that the live‐cell EV profiles obtained from neuronal cell cultures from three distinct brain tissue regions show a distinctive EV secretion fingerprint (Figure 6e), thus improving our understanding of specific vulnerabilities and local brain region differences that can be associated with the spread of neurodegenerative signals.

### 2.7 Okadaic Acid (OA) Treatment Results in Dysregulated miRNA Expressions Within Various Brain Regions in 9 DIV Neurons

**FIGURE 7:**
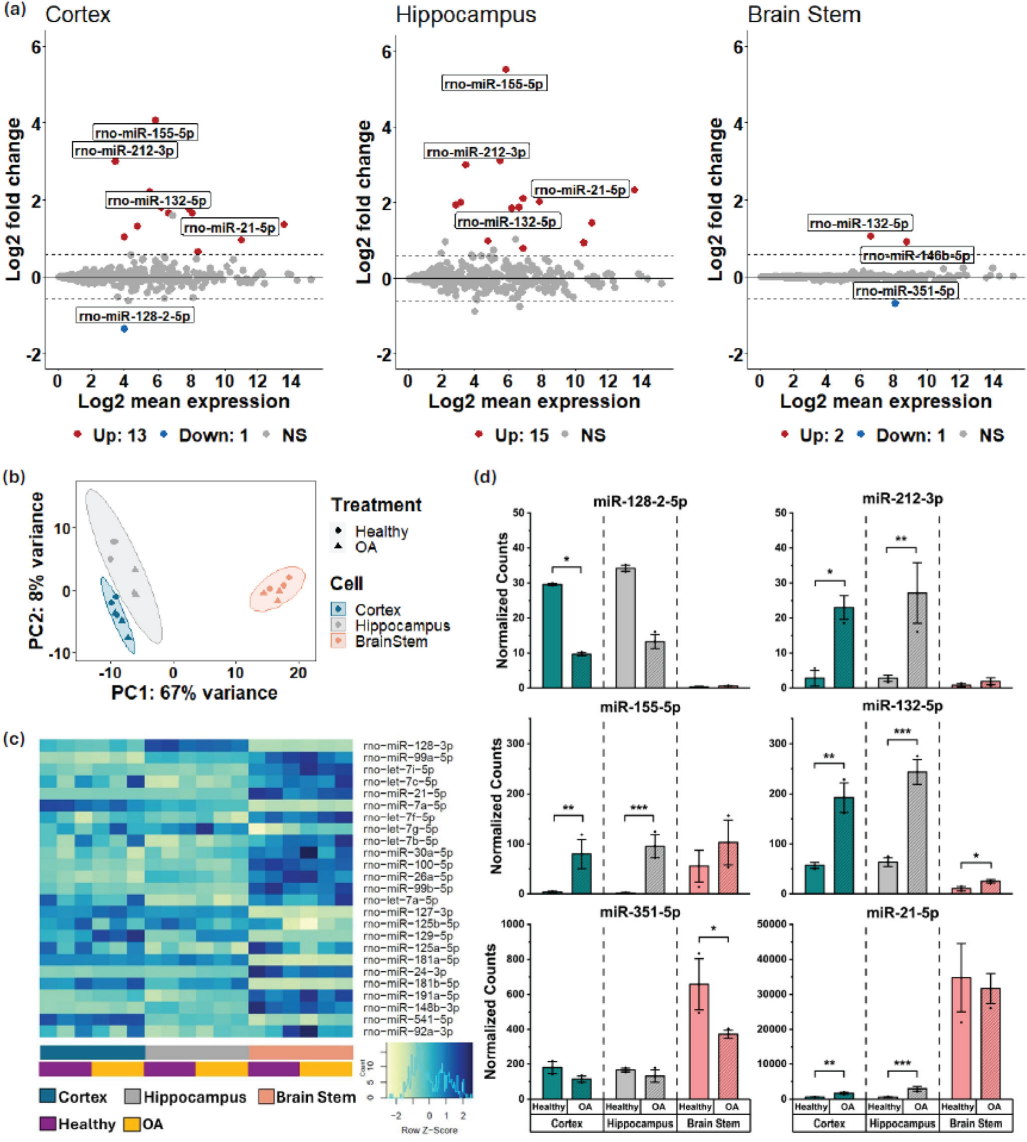
miRNA expression in whole cell neurons from RNA‐Seq. (a) Log2 fold change (LFC) versus Log2 mean expression for the miRNA in each cell type, with significantly upregulated miRNA in red and significantly downregulated miRNA in blue. A majority of the miRNA are not significantly dysregulated when comparing the OA‐treated cells to healthy controls (gray points). The ‘apeglm’ algorithm was used for LFC shrinkage, with thresholds of significance set at p_adj_ < 0.05, |fold change| > 1.5. (b) PCA comparison between different cell types and treatment conditions, with groupings determined at level = 0.95, α = 0.2. (c) Heatmap of the top 25 expressed miRNA in all samples. (d) Normalized counts of six selected miRNAs, comparing the OA‐treated samples to the healthy controls for each cell type (0.05> *p*_adj_ = ^*^; 10^−5^> *p*_adj_ = ^**^; 10^−10^> *p*_adj_ = ^***^).

As regulators of gene expression and intercellular signaling, miRNAs can indicate changes in cellular processes and health [58, 77]. We were interested in mapping miRNA responses to OA treatment to better connect the EV secretion rates to specific stress responses in neurons. To compare miRNA expression between healthy and OA‐treated neurons, we sequenced small RNA isolated from whole‐cell cultures of cortical, hippocampal, and brainstem rat neurons at 9 DIV. The OA‐treated neurons were collected 24 h after OA application. We found that cells cultured from the three different brain regions had approximately the same number of individually expressed miRNAs: 230 for cortex, 226 for hippocampus, and 230 for brainstem (Table S5). Most of the significantly expressed miRNAs are present in all three tissue regions. Interestingly, the next largest groups are miRNAs in the cortex and hippocampus and miRNAs only in the brainstem, indicating perhaps more similarities between the cortex and hippocampus. The brainstem also had the most uniquely expressed miRNA. For all tissue regions, the dysregulated miRNAs were mostly upregulated (Figure 7a, Table S6). PCA analysis did not find distinct groupings between healthy and OA‐treated cells, but this was expected because a majority of the expressed miRNA are not dysregulated (Figure 7b). Overall, the variation between samples is due to the cell cultures from different tissue regions, which can be visualized further by comparing the top 25 expressed miRNA across samples, where distinct patterns were observed between cell types but not between conditions (Figure 7c).

Of the dysregulated miRNAs, we selected miR‐21‐5p, miR‐128‐2‐5p, miR‐132‐5p, miR‐155‐5p, miR‐212‐3p, and miR‐351‐5p for further analysis based on a combination of prior association with neurological disorders and small‐EVs in the literature, and because of how these miRNAs were differently expressed between cell types in our study (Figure 7d). Three of these miRNAs were upregulated after OA treatment in the cortex and hippocampus but not in the brainstem: miR‐21‐5p, miR‐155‐5p, and miR‐212‐3p. miR‐21‐5p is a well‐established inflammatory biomarker implicated in many disease states, including cancer, cardiovascular diseases, and neurodegenerative diseases. MiR‐21‐5p was found to be upregulated in AD iPSC‐derived neurons and neuronal exosomes [78], as well as in several other AD models (3D organotypic hippocampal cultures transplanted with SWE cells, CSF of MCI‐AD patients, and AD iPSC‐derived astrocytes and microglia) [79]. However, miR‐21‐5p was downregulated in Aβ42‐treated primary hippocampal neurons and in the hippocampus of seven‐ and 13‐month‐old APP23 mice, when Aβ plaques were established [80]. MiR‐21‐5p was also downregulated in plasma‐derived EVs from Alzheimer's patients [81]. While miR‐21‐5p could be considered a non‐specific indicator of inflammation and stress because it is associated with many disease states, it is an important marker when combined with other miRNA and can provide insight into disease progression within different parts of the brain. Second, miR‐155‐5p has been shown to be upregulated in AD as a modulator of pro‐inflammatory responses within primary microglia, similar to our findings [82, 83]. Contrarily, miR‐212 has been shown to be downregulated in the temporal cortex of AD patients [84] and in neuronal exosomes collected from the plasma of AD patients [85]. Decreased levels of miR‐212‐3p lead to neuroinflammation through the SP1/BACE1 axis [86]. This is significant in how miR‐212‐3p is closely linked to another neurodegenerative marker, miR‐132‐5p. MiR‐132‐5p and miR‐212 are both on chromosome 17 of the human genome, and miR‐132‐5p was also downregulated in the temporal cortex [84] and in neuronal exosomes [85] during AD. Decreased levels of miR‐132 result in higher tau phosphorylation [87]. However, another study found exosomal miRNA ex‐miR‐132‐5p to be upregulated in the CSF of AD patients [88]. We also found that miR‐132‐5p was significantly upregulated in our cortical, hippocampal, and brainstem samples, which could indicate a neuroprotective response to the OA treatment [89]. Overall, our testing revealed elevated levels of miR‐21‐5p, miR‐155‐5p, and miR‐212‐3p (and the related miR‐132‐5p) in cortical and hippocampal 9 DIV neurons 24 h after OA treatment.

Lastly, we found miR‐128‐2‐5p to be significantly down‐regulated in the cortex (p_adj_ = 0.00593) and miR‐351‐5p to be significantly down‐regulated in the brainstem (p_adj_ = 0.0258). MiR‐128 down‐regulation is connected to an increase in tau phosphorylation through regulation of BAG2, an auxiliary chaperone protein that acts in the degradation and aggregation of tau [90]. MiR‐351‐5p was upregulated in the hippocampus of AD model mice and was shown to be involved in hippocampal neural progenitor cell death [91]. There are limited studies on miR‐351‐5p in the brainstem, specifically.

Notably missing from our findings is the dysregulation of several miRNA implicated in neurodegeneration related to tauopathies and amyloid‐beta regulation. MiR‐9 is an inflammatory biomarker that is dysregulated in extracellular vesicles and plays a role in tau regulation and synaptic function [92, 93]. MiR‐26b, miR‐34a, miR‐124, and miR‐922 are also involved in tau phosphorylation and pathology along various pathways [93]. MiR‐106a and b, miR‐29a, b, and c, miR‐107, and miR‐147 are associated with amyloid‐beta regulation [41]. We found no significant dysregulation in response to OA treatment for any of these miRNAs. Furthermore, we assessed the impact of the OA treatment on the formation of reactive oxygen species (ROS) and found no increase in ROS activity through the OA treatment (Figure S8). Overall, our findings align with a portion of the current research into miRNA expression with neurodegeneration, but there are important differences. These discrepancies highlight the need for standardized protocols to ensure careful sample preparation to eliminate RNA from sources besides the cell or exosome and consistency in RNA isolation and sequencing. The differences between our results and the literature could also point to the need for more information about disease progression with time and spatial specificity. Complete disease characterization will require a richer understanding of how miRNAs are dysregulated over time and within different brain tissue regions, subcellular locations, and EVs.

## 3 Conclusion

In this study, we investigated differences in subcellular EV size profiles in neurite networks of primary cortical, hippocampal, and brainstem cell cultures using a compartmentalized neurofluidic platform. Through EV isolation, cryo‐EM characterization, and DLS measurements, we refined EV profiling by reporting distinct relative changes in exomeres, exosomes, microvesicles, and apoptotic cell body‐sized extracellular nanoparticles between different culture growth platforms (Petri dish vs. neurofluidic) and exogenous modulations such as fluid shear forces and biochemical reagents in culture. The refined EV profiling method was then applied to extract a differential secretion pattern between somatic and neurite/axonal network compartments in our neurofluidic device. We found that subcellular axonal compartments differentially secrete EVs, and these profiles may depend on cell origin and cell culture composition. Next, we demonstrated that modulating cell signaling with a phosphatase inhibitor in different culture types results in distinct EV secretion profiles. We were also able to unravel each subtype of EV dynamics under this biochemical modulation using our neurofluidic device, which provided us with a higher‐resolution monitoring platform. We have also shown that mechanical forces, such as shear forces, significantly affect the EV secretion dynamics in both Petri dishes and the neurofluidic platform. Furthermore, we examined selected miRNAs that are known to be dysregulated in neurological conditions and observed subtle differences across cell types in response to OA treatment, particularly in cortical and hippocampal neurons. This helped clarify the overall effects of OA on the neurons in this study and will inform future work on miRNA content in EVs tied to their secretion patterns.

## 4 Materials and Methods

### 4.1 Primary Cell Culture From Dissociated Brain Tissues

Cortical, hippocampal, and brainstem cells were isolated from distinct tissue sections of whole embryonic rat brains (E18, TransnetYX). Separated tissues were dissociated with Papain (10%(v/v), Carica Papaya, Fischer Scientific) with Hibernate E‐Medium at 35°C for 15 min following previously established protocols [42, 94]. 2 h before cell seeding, all Petri dishes and microchannels in the neurofluidic devices were coated with poly‐l‐lysine (0.0025% PLL, Thermofisher Scientific A3890401). Residual PLL was removed from the dishes and channels through gentle washing with phosphate buffer saline (PBS). After tissue dissociation, cortex, hippocampus, and brainstem cells were centrifuged and seeded at a cell concentration of 5 × 10^5^ cells/ml in Petri dishes, and 1 × 10^5^ cells/ml in microfluidic all three somatic culture well compartments of the neurofluidic devices. To ensure cell adhesion in neurofluidic devices, the devices were put in the incubator for 5–10 min after the initial seeding. Then the neurofluidic devices were covered with culture medium and put back in the incubator. Cells were maintained and grown at 37°C and 5% CO2, and the cell culture media were supplemented with Neurobasal culture medium (Invitrogen) with B‐27 (2% (v/v), Invitrogen), 1% GlutaMAX Supplement (Thermofisher Scientific, 35050061), and Pen Strep (1% (v/v)). Brainstem cells were supplied with Neurobasal Plus culture medium (Gibco, Thermo Fisher Scientific) with 10% Horse Serum (Gibco, Thermo Fisher Scientific). The culture medium was completely exchanged both in Petri dishes and neurofluidic devices every three days at (3,6,9) days in vitro (DIV).

### 4.2 EV Isolation

EVs were isolated from conditioned cell culture media at 3 DIV and 9 DIV using Total Exosome Isolation Reagent (Fisher Scientific) according to the instructions of the manufacturer. Briefly, constant volumes (500 µL) from 2 mL cell‐conditioned media were collected from Petri dishes after a gentle 5 s swirl to ensure a homogenous mixture of extracellular vesicles. The same volume of sample was collected from one of the culture wells and the perfusion channel in the neurofluidic devices. The samples were collected from a single somatic culture well and the entire perfusion channels. The samples were then centrifuged at 25°C, 300 x g for 10 min to remove floating cells and cell debris. The supernatant was transferred into a sterile, empty centrifuge tube and centrifuged at 25°C, 2000 x g for 30 min. After the supernatant was transferred to another sterile, empty centrifugation tube, it was mixed with the Total Exosome Isolation Reagent (1:2 reagent: supernatant ratio (v/v)) and incubated overnight at 4–6°C. The next day, the mixture was centrifuged at 10 000 x g for 60 min at 4°C, and the supernatant was discarded. After discarding the supernatant, the debris containing EVs was resuspended in nuclease‐free PBS (50 µl). After 2–5 days at 4°C, EV/PBS resuspensions were stored at −80°C for the long term.

### 4.3 EVs Size Profiling and Post‐Data Aanalysis

Dynamic Light Scattering (Mobius, Wyatt Technology) was performed to extract an EV size profile across a hydrodynamic radius range of 15 nm to 5 µm. After thoroughly mixing the 50 µL of EV/PBS resuspension, 10 µL (20%) of the sample was used and diluted in PBS (1:50) and subsequently analyzed using NTA at 25°C with a detection angle of 163.5° at 532 nm laser wavelength. The raw EV particle counts were then exported and normalized by the collection volume, initial plated cell density, and daily particle secretion rates. Due to the radius ranges that were chosen for each subcategory of the EVs, between the exosome (r = 25–75 nm) and microvesicle counts (r = 50–500 nm), the radius of (r = 62.443 nm) from the DLS raw readings was counted twice. The rest of the EV/PBS suspension was stored at ‐80°C.

### 4.4 Cryo‐Electron Microscopy Sample Preparation and Imaging

The samples were plunge‐frozen in precooled liquid ethane using Vitrobot Mark IV (FEI, USA) plunge freezer set at 5°C and 95% humidity. 4 µL of sample was pipetted onto a Quantufoil 200 mesh R2/1 Cu grid and blotted for 4 s at a blot force of 3 with cellulose blot paper on both sides. The frozen grids were then imaged on a Talos Arctica transmission electron microscope operating at 200 kV. Images were recorded with a Gatan K3 camera using SerialEM at a magnification of 36 000x, and at −3 µm defocus [95]. Exposure times were 3 s at approximately 1 electron per pixel per second and a total dose of 26 e^−^/Å [2].

### 4.5 Protein Hyperphosphorylation With OA

Okadaic acid (OA), a protein phosphatase 1A (PP1A) and a protein phosphatase 2A (PP2A) inhibitor, was dissolved in DMSO and diluted in neuronal culture medium down to 100 nM. To test if 100 nM of okadaic acid induced any reactive oxidative stress in rat primary neurons, we looked at the ROS expression in cortical neurons (Figure S9). At 100 nM, we see no significant changes in cell ROS expressions.

At 9 DIV, cortical, hippocampal, and brainstem cell cultures were incubated with OA for 60 min in Petri dishes or the three somatic wells in the neurofluidic cultures. Subsequently, all cultures were gently washed with pre‐warmed culture media and further imaged. Before and after the OA treatment (pre, 12, and 24 h), cell‐conditioned media were collected to purify EVs.

### 4.6 Live Cell Imaging

To capture cell density, cell morphology, the maturity of the neurite network, and signs of neurodegenerative events in the neurite network, live‐cell imaging was performed using an inverted microscope (Leica DMi‐8) with 20X and 10X air objectives and differential interference contrast (DIC). Cell cultures were imaged before and after EV isolation, and stained for reactive oxygen species (ROS, Figure S8), Fluo4‐AM, and DiI lipid staining following vendor protocols.

### 4.7 Neurofluidic Device Design and Fabrication

To extract EV profiles from subcellular compartments, we designed a neurofluidic device in CleWin. The device comprises three somatic wells, which are connected to a peripheric microfluidic channel through radially arranged junction channels (Figure 5a). The junction channels are 5 µm high with varying lengths between 557 and 1353 µm, keeping the channels at an equal hydrodynamic resistance. The final design was then transferred to transparency masks for cleanroom fabrication. In the clean room, a neurofluidic device master is fabricated based on a two‐step photolithography process using KMPR1005 for the 5 µm tall junction channel features and KMPR1050 (MicroChem) for the 50 µm tall somatic well and perfusion channel features. Spin coating speed parameters (100 rpm/s to 4000 rpm for 30 s) were adjusted to ensure air bubble‐free coating of the two photoresist layers without impacting photoresist thickness. Figure S9 shows a schematic of the clean room wafer fabrication processes in detail. After master fabrication, microchannels were cast into polydimethylsiloxane (PDMS, Dow Corning Slygard) and cross‐linked in a 10:1 ratio at 65°C for 2.2 h. After gently peeling off the PDMS slab, the mold was cut into 1 cm x 2 cm devices, and the three somatic compartments were punched open (Ø 4 mm). Next, all PDMS devices were autoclaved (120°C, 2 h 12 min). Next, unsealed microchannels were closed through plasma bonding the PDMS devices to glass‐bottom slides. All microchannels and side walls were coated with PLL before cell culture to ensure neuronal adhesion in culture wells. EV diffusion times were calculated for the shortest and the longest junction channel lengths (Table S1). We further extended our analyses and showed the flux and the percentage of our smallest EVs diffusing through the shortest junction channel. The calculations are shown in the Supporting Information.

### 4.8 RNA Isolation From Neurons

The Zymo Quick RNA Microprep Kit, a spin column‐based tool, was used to extract RNAs (greater than 17 nucleotides) from whole‐cell neurons. Briefly, cortical, hippocampal, and brainstem neurons were cultured in Petri dishes (n = 4 for each cell type + treatment/control). At DIV9, 100 nM okadaic acid was applied to each treatment sample, incubated for 1 h, and then replaced with culture media. After 24 h, the cells for all samples (treatment and healthy control) were lysed by removing the culture media from each pipette dish and adding 300 µL of RNA lysis buffer to each dish. The lysed cells were transferred into tubes, spiked with 2 µL cel‐miR‐39‐3p Spike‐in Template (Qiagen, ID. 339390), and processed according to the Zymo Quick RNA Microprep instructions, including the on‐column DNAse treatment. To remove residual organic contamination, an additional wash step and a final dry centrifugation step were added. RNA was eluted in 25 µL nuclease‐free water. RNAs were quantified using a Nanodrop 1000 Spectrophotometer with the nucleic acid application module and Sample Type “RNA”. The instrument was blanked with 1 µL nuclease‐free water. Each sample was quantified three times to verify accuracy. The pedestal was cleaned with nuclease‐free water between each measurement. The replicate from each sample with the highest RNA yield was selected for further sequencing.

### 4.9 Small RNA Library Preparation and Sequencing

Small RNA sequencing was conducted by the University of Montana Genomics Core (Missoula, Montana). RNA libraries were prepared using the NEBNext Small RNA Library Prep Set for Illumina (New England Biolabs, Ipswich, MA, USA). Fragments were analyzed for quality and size distribution using the Agilent TapeStation system with D1000 ScreenTape. Fragments were size selected for 133 to 203 bp, which corresponded to any fragment insert from 10 to 80 bp (123 bp primer/adaptor dimer). Each library was barcoded and split into two pools with equal molarity (Pool 1 = healthy samples 1–9; Pool 2 = treatment samples 10–18). Paired‐end sequencing was done using the MiSeq Reagent Kit v3  for Illumina with 75 cycles, resulting in about 2.5 million reads per sample.

### 4.10 Analysis of Small RNA Sequencing Data

A summary list of the software and databases used in the bioinformatics pipeline is detailed in Table S2. Briefly, Cutadapt was used to trim the adaptors and to remove reads shorter than 18 bp and longer than 32 bp to retain reads in the length range of mature miRNA (22–25 bp) [96]. Trimmed reads were aligned to the Ensembl rat genome (Rnor 6.0) using Bowtie 1, allowing for paired‐end alignment with pairs that overlap the same reference interval with the “–allow‐contain” option (Table S3) [97]. MiRNA counts were generated from the miRbase rat annotation file (rno.gff3) using featureCounts [98]. Differential expression analysis was done using DESeq2 (v1.42.1) [99]. Specifically, the raw counts were normalized for library size using the DESeq2‐calculated size factors (Table S4; Figure S10). MiRNA were considered significantly expressed if at least three samples had a normalized count greater than or equal to 5 (Table S5). The differential expression between healthy and OA‐treated cells for each cell type is calculated by DESeq2 using a negative binomial distribution. The threshold for differentially expressed miRNA was set at a padj ≤0.05 and a fold change of ≥ 1.5 (Table S6). MA plots, PCA plots, heatmaps, and miRNA scatter plots were created using the ggpubr (v0.6.0), gplots (v3.1.3.1), and ggplots2 (v0.6.0) packages in R Studio.

## Author Contributions

Z.M. performed device fabrication, cell cultures, exosome isolation, data analysis, experimental design, figure artwork, and writing of the manuscript. ES performed experimental design for micro‐RNA isolation, data analysis, figure artwork, and writing of the manuscript. S.E.M. performed experimental design and supervision, provided reagents, and wrote the manuscript. A.K. performed device design, fabrication supervision, experimental design supervision, data analysis, figure artwork, and writing of the manuscript. All authors read, revised the manuscript critically, and approved the final version of the manuscript.

## Funding

This work was supported through a Thornson Excellence in Engineering Research (TEER) Grant, Montana State University Catalyst Gap Fund (Grant# ED19HDQ0200091 and NIA #1R21AG071691‐01), and the National Science Foundation (CAREER Grant #1847245). Partial work was performed at MMF, which belongs to the Montana Nanotechnology Facility (MONT), a member of the National Nanotechnology Coordinated Infrastructure (NNCI) supported by the National Science Foundation (Grant# ECCS‐1542210). Funding for the Montana State University Cryo‐EM Core Facility (RRID: SCR_026324) was contributed by the National Science Foundation (DBI‐1828765), the MJ Murdock Charitable Trust, the National Institute of General Medical Sciences (P30GM140963), and the MSU Office of Research, Economic Development, and Graduate Education. This research was also supported by the University of Montana Genomics Core and Montana INBRE Data Science Core, which are funded by the National Institute of General Medical Sciences (P20GM103474), the Office of the Vice President for Research and Creative Scholarship at the University of Montana, and the M. J. Murdock Charitable Trust. The content is solely the responsibility of the authors and does not necessarily represent the official views of the UMGC or the National Institutes of Health.

## Conflicts of Interest

The authors declare no conflict of interest.

## Supporting information




**Supporting File**: adbi70097‐sup‐0001‐SuppMat.docx.

## Data Availability

The data that support the findings of this study are available from the corresponding author upon reasonable request.
