# Supplementary material for: Sub‐Neuronal Network Profiling of Extracellular Vesicle Release Using a Compartmentalized Neurofluidic Platform
Source: Adv Biol (Weinh). 2026 Feb 18;10(2):e00381. doi: 10.1002/adbi.202500381 (PMC12914630; doi:10.1002/adbi.202500381)
Supplement: Supplementary file 1 — Supporting File: adbi70097‐sup‐0001‐SuppMat.docx. [file ADBI-10-e00381-s001.docx]

**SUPPLEMENTARY DATA and INFORMATION for:**

**SUB-NEURONAL NETWORK PROFILING OF EXTRACELLULAR VESICLE RELEASE USING A COMPARTMENTALIZED NEUROFLUIDIC PLATFORM**

Zeynep Malkoc, Esther Stopps, Prince M. K. Asamoah, Stephanie E. McCalla, Anja Kunze*
*Corresponding author: Dr. Anja Kunze; Email: anja.kunze@montana.edu

Ms. Zeynep Malkoc:
Department of Chemical and Biological Engineering, Montana State University, Bozeman, Montana, 59717, USA.

Ms. Esther Stopps:
Department of Chemical and Biological Engineering, Montana State University, Bozeman, Montana, 59717, USA.

Mr. Prince M. K. Asamoah:

Department of Chemistry and Biochemistry, Montana State University, Bozeman, Montana, 59717, USA.

Prof. Dr. Stephanie E. McCalla:
Department of Chemical and Biological Engineering, Montana State University, Bozeman, Montana, 59717, USA.

Prof. Dr. Anja Kunze:

Department of Electrical and Computer Engineering, Montana State University, Bozeman, Montana,59717, USA.
Montana Nanotechnology Facility, Montana State University, Bozeman, Montana,59717, USA.

**Abstract:**

This document provides further details about the main article mentioned above. Additionally, supplementary graphs, calculations, and raw data are included to support the results described in the main article. This document features sixteen total supplementary figures, S1 – S10, and tables S1- S6.

**Verification of Dynamic Light Scattering (DLS) measurement sensitivity**

To verify the sensitivity of Dynamic Light Scattering (DLS) to the concentration changes in the samples, we ran control samples using starch-amine-coated magnetic nanoparticles with a radius of 50 nm. The samples had concentrations of 6x10^6^ nanoparticles/ml, and 3x10^7^ nanoparticles/ml. These verification measurements demonstrated that the relative number of particles detected by the instrument is quantitatively sensitive to the changes in particle concentration present in the samples (Figure S1a-d).

| **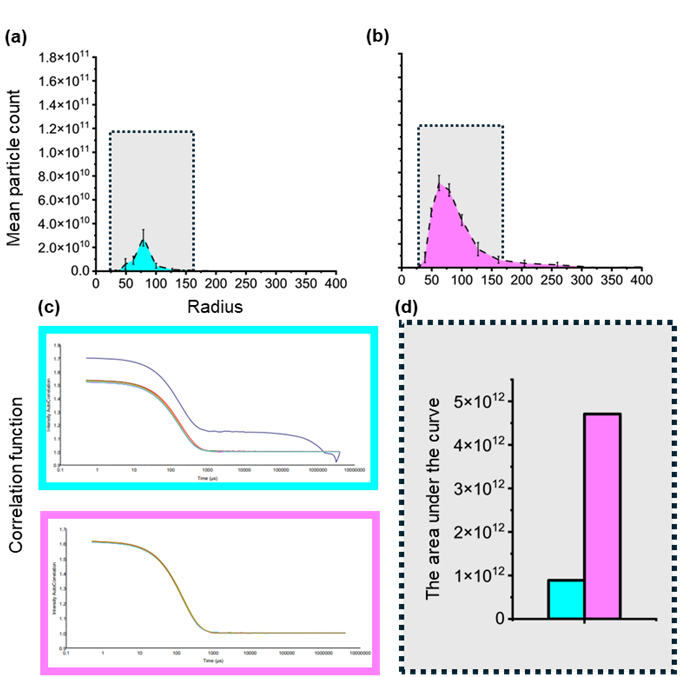** |
| --- |
| Figure S1: Verification of DLS measurement sensitivity to particle number by measuring two concentrations of 50 nm radius starch-amine coated magnetic nanoparticles. The relative mean particle count comparison of magnetic nanoparticles with concentrations of (a) 6x10^6^ nanoparticles/ml , and (b) 3x10^7^ nanoparticles/ml. (c) Correlation functions extracted from the DLS readings. (d) The area under the curve seen in part a was also calculated to show the total relative number of particles counted via DLS. As expected, five times the concentration of particles produced five times value for the area under the curve. |

**Efficacy assessment for the extracellular vesicle extraction kit**

To evaluate the effectiveness and performance of the total exosome isolation reagent (TEIR), we conducted dynamic light scattering (DLS) on samples collected from the same Petri dish, processed with different buffers (n = 3…5). The first samples were collected from cortical cells and processed overnight with PBS instead of TEIR. We then compared the results of the DLS analysis with media samples processed with the TEIR. Figure S2a and Figure S2b1-b2 show the different particle counts for each EV class and experimental condition extracted with (w/kit) and without (no kit) the TEIR EV extraction kit. We observed a significantly higher particle secretion rate with the small extracellular vesicles (sEVs, exomeres and exosomes) with TEIR (w/kit), which indicates that the isolation kit has higher recovery rates with these particles. We did not note any significant differences with microvesicles and apoptotic cell bodies, which were co-isolated in both conditions.

| **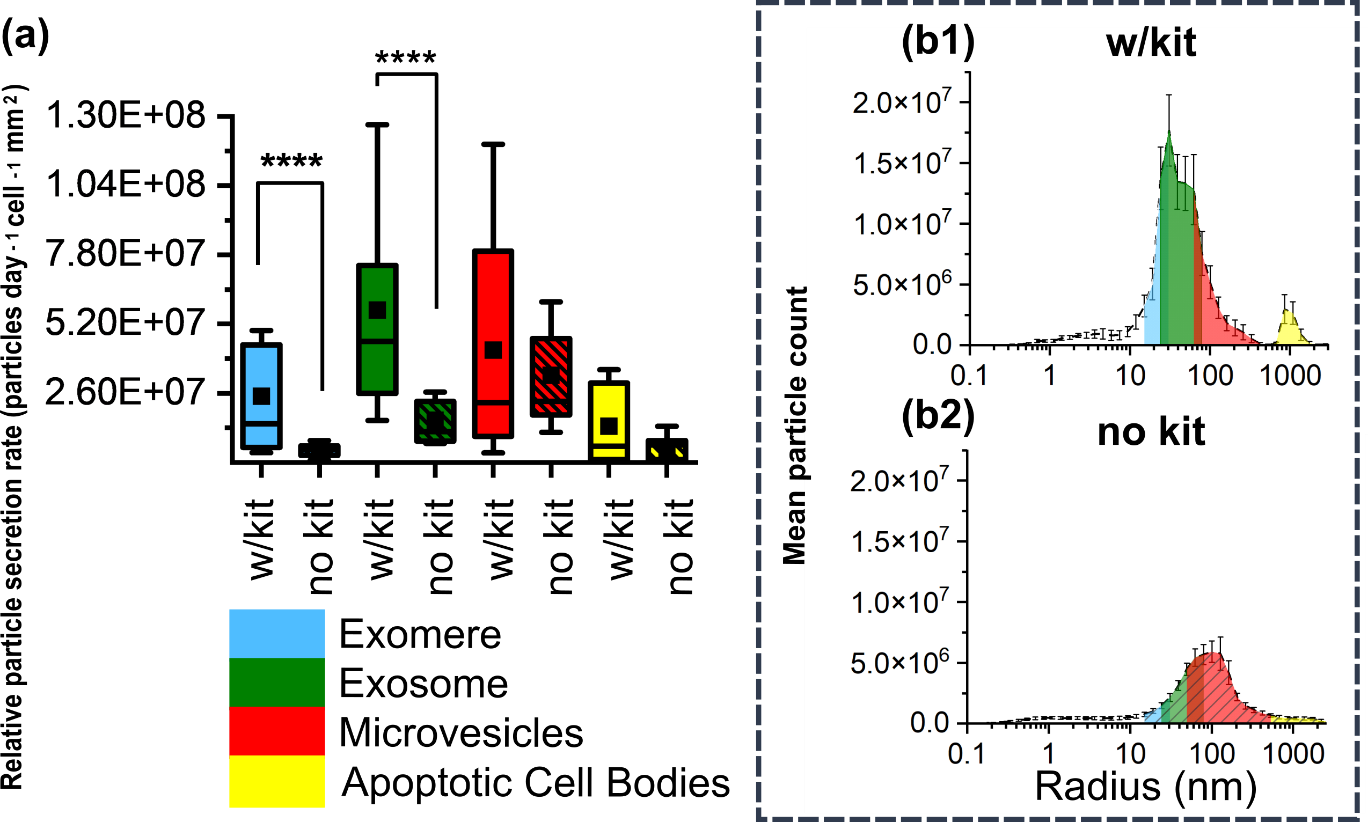** |
| --- |
| Figure S2: Verification of extracellular vesicle extraction efficiency. To test the robustness and the extraction efficacy, the same extraction steps were followed with the conditioned media, in the presence of the total exosome isolation kit (w/kit) and using PBS instead of the kit (no kit). (a) Box plot reveals that the exomere and exosome recovery is significantly higher when the total exosome isolation kit is used. The kit has not shown any significant efficiency with the recovery of microvesicles. A non-parametric Mann-Whitney U test was conducted to compare the data at a significance level of α=0.05, where ****p<0.0001. (b1) Mean particle counts are shown for each EV subtype of the samples subjected to TEIR, n >3 (b2) Mean particle counts are shown for each EV subtype of the samples subjected to PBS only, n >3 |

**Different sample dilutions show similarities in EV size profiling, indicating stable EV populations without aggregation artifacts**

To verify that our DLS-based quantification method is minimally influenced by concentration-dependent scattering or reversible aggregates, we analyzed paired samples collected at 3 DIV at two independent dilutions, 1:50 (as used in our methods) and 1:100 (Figure S3a1-c2). If strong aggregation effects were to occur, we would expect a decrease in radius size measurements with higher dilutions. Across all the brain tissue regions (cortex, hippocampus, and brainstem), particle distributions were overlapping across the dilution sets, with normalized mean relative particle count rates of both dilutions showing a peak at a range of 50-200 nm in radius.

| **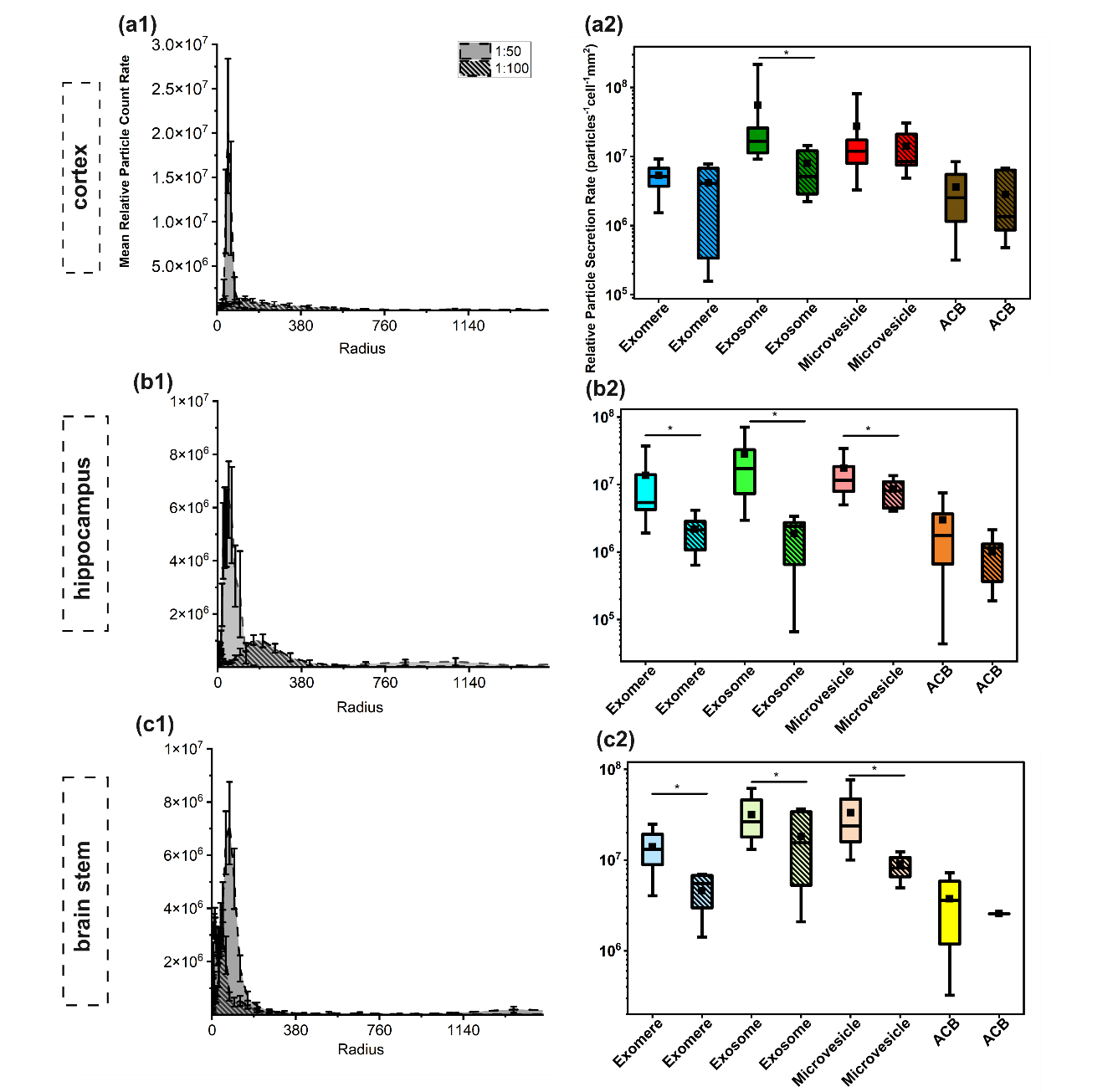** |
| --- |
| Figure S3: Normalized mean relative particle count rates for EVs isolated from (a1) cortex, (b1) hippocampus, and (c1) brainstem, measured at two independent dilutions (1:50, non scattered; 1:100, scattered). Box plots on the right show corresponding size-binned relative secretion rates across biological replicates for each brain region: (a2) cortex, (b2) hippocampus and (c2) brainstem. A non-parametric Mann-Whitney U test was conducted to compare the data at a significance level of α=0.05. 1:50 dilution sets had n_biological_ = 5 biological replicates, and 1:100 dilution sets had n_biological_ = 3 biological replicates for this study with N_measurement_ = 7 measurement replication on DLS. |

**Morphological changes are shown in healthy neuronal cultures through bright-field imaging**

Our study investigated EV secretion dynamics from three different brain tissue regions: the cortex, hippocampus and brainstem (Figure S4a-c). We collected the EV samples from cell culture media every three days (3 DIV, 6 DIV, 9 DIV), and did a total media change after sample collection. Bright-field images of cells from these three regions show the establishment of networks between the neurons as the cells get closer to maturation. By 9 DIV, cortex, and hippocampal neurons grow dense neurite networks in comparison to younger neurons at 3 DIV. The brainstem shows a higher growth of supporter cells (glia and astrocytes) between 3-6 DIV.

| **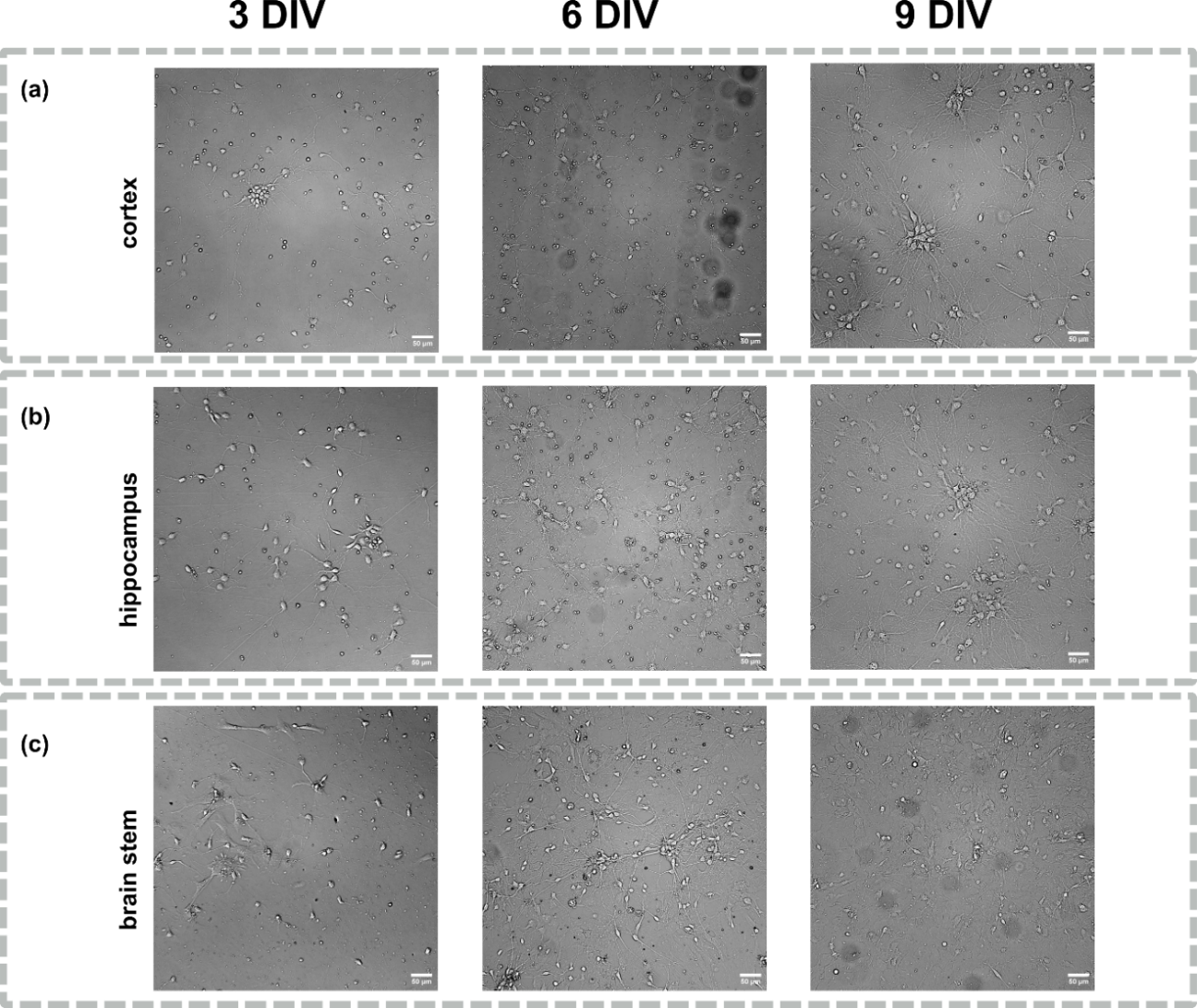** |
| --- |
| Figure S4: Neuronal cultures from three distinct brain regions’ (a) cortex, (b) hippocampus, and (c) brainstem. Bright-field images are shown at 3 DIV, 6 DIV and 9 DIV. Scale bar = 50 µm. |

**Cell morphology phenotypes of tissue-specific brain cell cultures and changes under**

**okadaic acid (OA) treatment**.

All three cell culture types (cortex, hippocampus and brainstem) were exposed to a 1 h okadaic acid (OA) treatment and further imaged after gentle washing. Our OA *in vitro* disease model is used a protein phosphatase-2A inhibitor^1-5^. This inhibitor is known to induce tau hyperphosphorylation, beta-amyloid deposition, and neuronal death, which are some of the characteristic features of Alzheimer's Disease (AD)^3^. Tau hyperphosphorylation leads to the formation of neurofibrillary tangles and increases reactive oxidative stress (ROS)^6, 7^, resulting in cellular neurotoxicity. The OA treatment led to neurite degeneration as described in other studies. Figure S5a-c shows the morphological changes in cortical, hippocampal, and brainstem neurons on images taken before treatment and 24 hours after OA treatment.

| 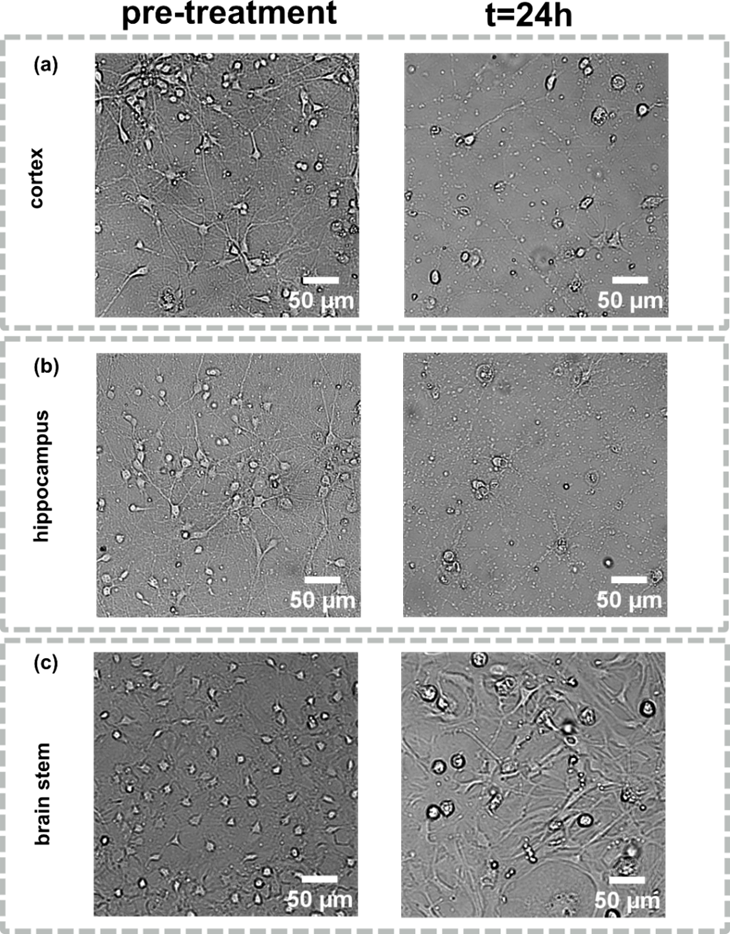 | | |
| --- | --- | --- |
| Figure S5: Morphological changes in the three tissue cultures after okadaic acid (OA) treatment in (a) cortex, (b) hippocampus, and (c) brainstem. Scale bar = 50 µm. |  |  |

**Mechanical forces caused by frequent pipetting and media change during the OA treatment protocol can shifts EV secretion rates**

To control for the potential effects of the experimental protocol itself while we perform OA treatment (Figure S6a-b), we performed an additional set of control experiments. The “pre” control consisted of samples collected from the cell cultures at 9 DIV, immediately before any media change or reagent addition. These “pre” samples represent the baseline EV secretion rate before any type of experimental manipulation takes place (Figure S6c-f). The “sham” control group underwent the same experimental protocol as the OA treatment group; but with unconditioned culture media instead of OA-conditioned media. By comparing the EV secretion rates in the ”sham” controls to the “pre” controls, we aimed to isolate any effects of the frequent media change procedure and its correlated effect on EV secretion rates. In Petri dishes the frequent pipetting indeed had a significant effect on the EV secretion rates. There was a significant particle release increase recorded 12 hours and 24 hours after the sample collections from the “sham” control group when compared with the “pre” baseline control. The only exceptions to this trend were the 24-hour sham group’s unchanged microvesicle secretion rate in the hippocampus (Figure S6), the 12-hour apoptotic cell body secretion rate in the hippocampus (Figure S6), and the overall apoptotic body secretion rates in the brainstem (Figure S6). Despite some non-significant results, overall, we can confidently say that frequent pipetting may significantly increase EV secretion rates for many sub-EV types in the cortex, hippocampus, and brainstem in the Petri dishes.

| 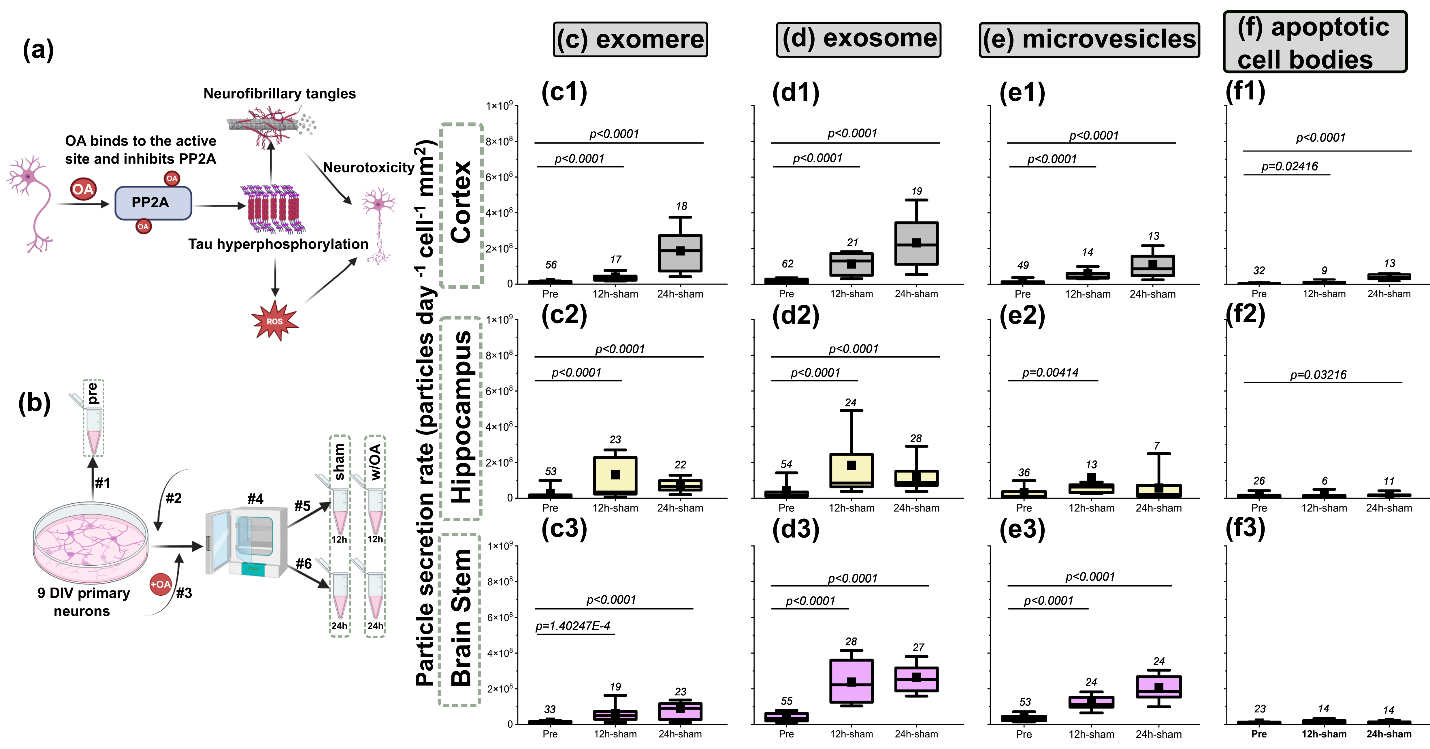 |
| --- |
| Figure S6. EV secretion rates under OA-free treatment. (a) Schematic diagram of the *in vitro* disease model showing the use of okadaic acid (OA) as a protein phosphatase-2A inhibitor. (b) Process flow: Pre-treatment samples were collected from Petri dishes prior to the application of OA treatment at 9 DIV. (#1) The sham group were subjected to a fresh unconditioned media to be used as a baseline (#2), and treatment groups were subjected to 100 nM OA treatment and incubated for 1 hour (#4). After the incubation period, the cells were washed and provided with fresh culture media. Subsequently, samples were collected at 12 hour (#5) and 24 hour intervals following the washing process (#6). Figure S5 compares the “pre” samples with the “sham” group to account for effects of mechanical forces due to frequent pipetting during the OA treatment experiments. Figure S5 (c-f) shows EV secretion rates after the mechanical exogenous forces in Petri dishes. These control experiments were carried out in will cell cultures from the three different brain regions: cortex (c1-f1), hippocampus (c2-f2), and the brainstem (c3-f3). A non-parametric Kruskal-Wallis ANOVA test was conducted with a significance level α=0.05 to compare all the experimental groups for each tissue region and EV subtypes. The numbers given above the boxplots are the experimental replicates. (a) and (b) were created in BioRender. Malkoc, Z. (2025) https://BioRender.com/rlgiqct. |

**Comparative summary of EV subtype dynamic responses to okadaic Acid (OA) treatment across brain tissue regions in the neurofluidic devices and Petri dishes**

We show a summarized visualization of region and sub-neuron compartment-specific effects of OA treatment on EV secretion dynamics in neurofluidic devices and in the Petri dishes. Figure S7 highlights how OA treatment leads to distinct EV subtype secretion responses across the cortex, hippocampus and brainstem. Overall, we noted a decrease in secretion rates of small EVs (exomeres and exosomes) in cortical neurons after OA treatment. Hippocampal neurons showed a response at the earlier time point, 12 h both in Petri dishes and mostly at the culture well of the neurofluidic devices. The brainstem response was more muted in the Petri dishes, whereas the dynamics were changing effectively at the peripheric channels of the neurofluidic device. Overall, these results suggest that our neurofluidic platform is a powerful tool to map both regional and compartmental dynamic changes in EV-mediated signaling under pathological stress.

| **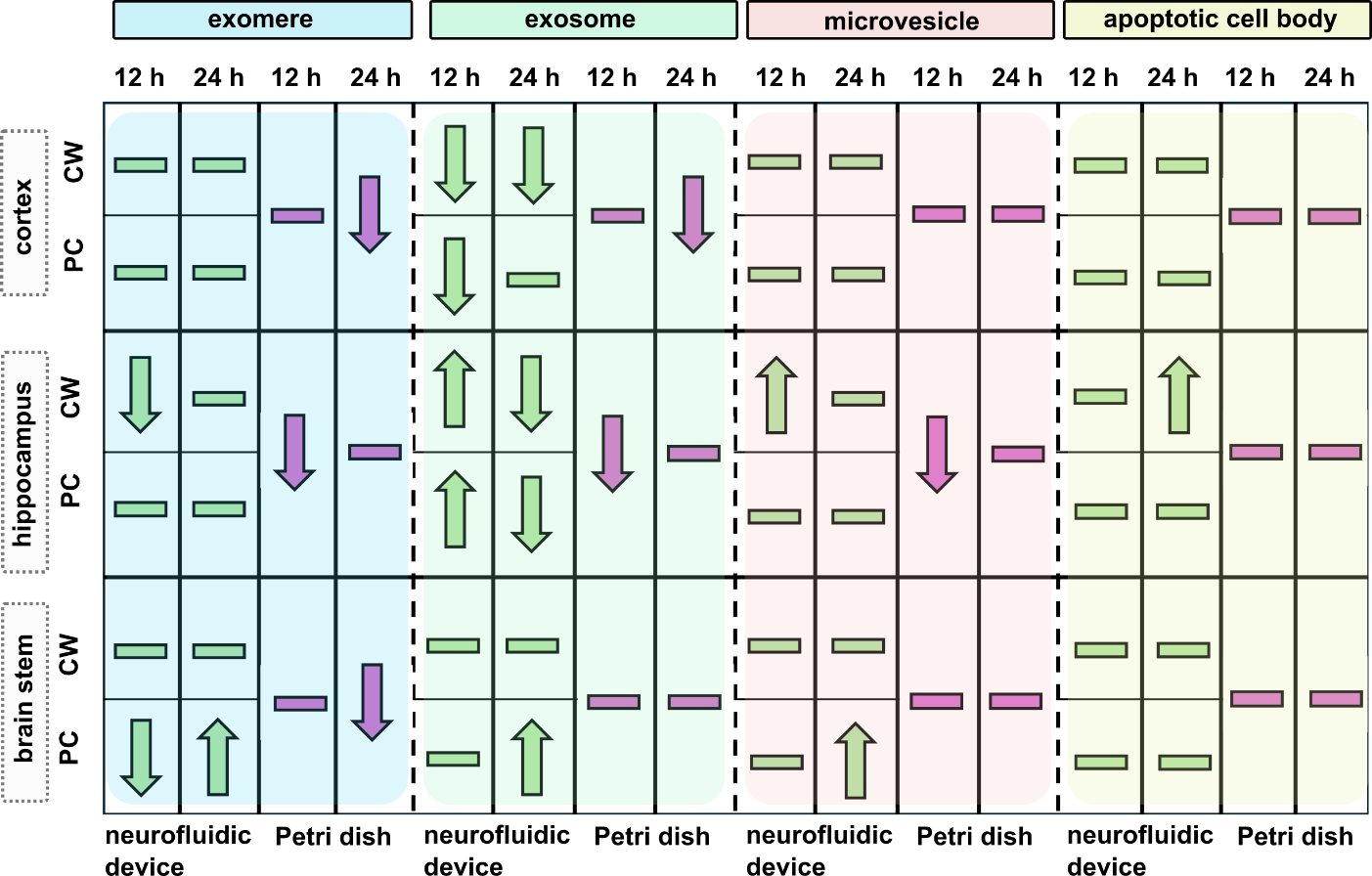** |
| --- |
| Figure S7. Schematic outlines the relative secretion rate changes in secretion of the EV subtypes: exomeres, exosomes, microvesicles, and apoptotic cell bodies. The dynamics are mapped across the three neuron types, cortex, hippocampus, and the brainstem, after 12 and 24 h of okadaic acid treatment. The trends for the secretion rates are represented across both compartments of neurofluidic devices (shown in green symbols): culture well (CW) and peripheric channel (PC), and Petri dishes (shown in purple symbols). Arrows indicate relative increases and decreases in the secretion rates, while dashes indicate no significant changes in EV secretion rates. |

**No significant changes in cell reactive oxidative stress were recorded 12 h and 24 h after okadaic acid treatment in primary rat cortical neuron cells**

In this experiment, we aimed to test if the EV profile dynamics with okadaic acid treatment resulted from a reactive oxidative stress-dependent pathway. Cortical cells at 9 DIV were exposed to okadaic acid with a concentration of 100 nM and were incubated for 1 h. After incubation, the cells were gently washed, and the media was replaced with fresh cell culture media. ROS indicator was added to the cells 30 min before the wash. Control groups included cells expressing ROS in their native state without any treatments ( +ROS, -OA), and cells with no ROS indicator and OA treatment (-ROS, -OA, Figure S8a). All cells were imaged 12 h and 24 post-wash. From each Petri dish, 3 to 5 images were taken, and from each image 25 to 50 cells were selected for analysis. This process was repeated across three biological replicates (n = 3) per experimental group. The intensity of each cell recorded was normalized to the background intensity of the selected region of interest (ROI). Data were then averaged for each Petri dish, and the results represent the average normalized fluorescent intensity values per dish across the replicates (Figure S8b). The normalization of the intensity is shown in Eq. 1:

*Normalized Fluorescent Intensity* =$\frac{Fcell-Fbackground}{Fbackground}$ (Eq. 1)

| **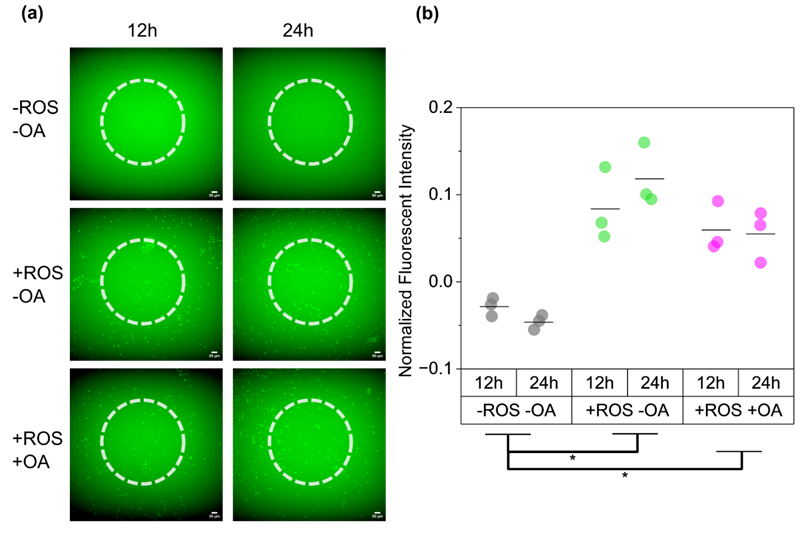** |
| --- |
| Figure S8: No significant differences in reactive oxidative species (ROS) levels were observed in primary rat cortical neurons. (a) 3 control groups included cells with no ROS indicator and no OA treatment (-ROS -OA), with ROS indicator and no OA treatment (+ROS -OA), and with ROS indicator and OA treatment (+ROS +OA) (scale bar = 50 µm). The same ROI was selected across the images for data analysis shown in a white circle. (b) Data shows the normalized fluorescent intensity across the 3 experimental groups. Each data point represents an average fluorescence intensity of individual cells from stacks of images (n_images_=3-5) taken from each biological repetition (n = 3). 2-way ANOVA was performed with a significance level of α = 0.05. |

**Neurofluidic device fabrication process**

The neurofluidic device master is fabricated based on a two-step photolithography process shown in Figure S9. The process flow uses KMPR1005 for the 5 µm tall junction channel features and KMPR1050 (MicroChem) for the 50 µm tall somatic well and perfusion channel features. Spin coating speed parameters (100 rpm/s to 4000 rpm for 30 s) were adjusted to ensure air bubble-free coating of the two photoresist layers without impacting photoresist thickness. After master fabrication, microchannels were cast into polydimethylsiloxane (PDMS, Dow Corning Slygard) and cross-linked in a 10:1 ratio at 65°C for 2.2 h. After gently peeling off the PDMS slap, the mold was cut into 1 cm x 2 cm devices, and the three somatic compartments were punched open (Ø 4 mm). Next, all PDMS devices were autoclaved (120°C, 2 h, 12 min). Next, unsealed microchannels were closed through plasma bonding the PDMS devices to glass bottom slides. All microchannels and side walls were coated with PLL before cell culture to ensure neuronal adhesion in culture wells.

| **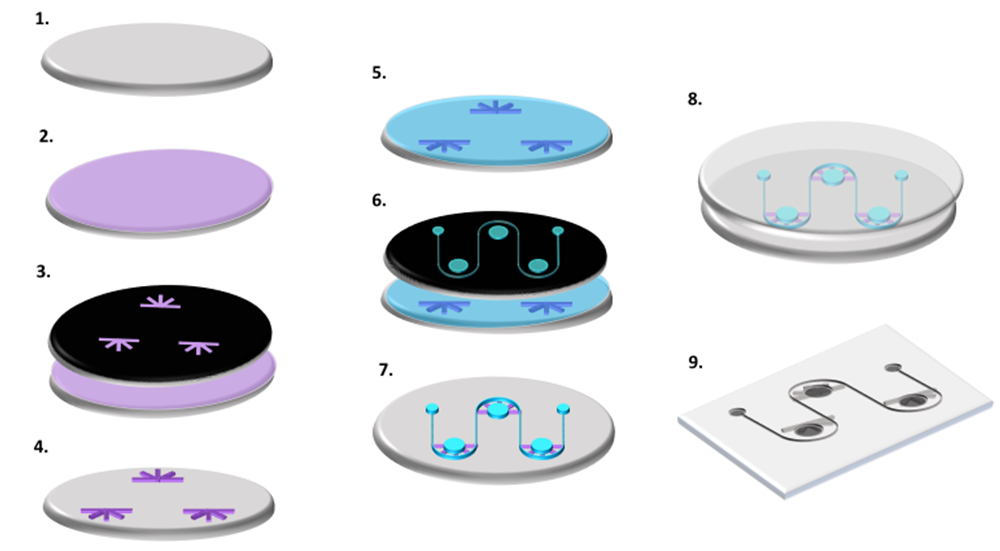** |
| --- |
| Figure S9: Cleanroom process flow shown in steps: 1,2: The silicon wafer is covered with KMPR1005 (purple) as the first photolithography step. 3,4: The mask for junction channels was then exposed to create the first layer of the neurofluidic platform. 5: Second layer of KMPR 1050 for peripheric channels poured on the same silicon wafer. 6,7: The mask for the peripheric channels were aligned and exposed to fabricate the final layer of the neurofluidic platform. 8,9: Once the master wafer is fabricated, the neurofluidic platform was cast into polydimethylsiloxane (PDMS) for further experimental use. |

**EV diffusion properties through junction channels**

Based on a two-dimensional Stokes-Einstein Equation diffusion times for the specific particle sizes were computed. Based on the minimum (557 µm) and maximum (1353 µm) length within the microchannel system, which connects the cell body well with the perfusion channel, maximum and minimum diffusion times are shown in Table S1.

**Table S1: Junction channel parameters and EV diffusion properties**

| Channel Length | Exomeres  R_avg_= 20 nm | Exosomes  R_avg_= 50 nm | Microvesicles  R_avg_= 275 nm | Apoptotic Cell Bodies  R_avg_= 1500 nm |
| --- | --- | --- | --- | --- |
| 557 µm | 2.6 hours | 6.5 hours | 22 hours | 197 hours |
| 1353µm | 15.5 hours | 38.7 hours | 131 hours | 1161 hours |

Legend: R_avg_ … average hydrodynamic radius

To model the transport of EVs across the microchannels from the culture well to peripheric channels to ensure we do not have cross-contamination between the two wells, we adopted a quasi-steady-state approach, treating the microchannels as a simplified membrane. This assumption is valid given the relatively slow changes in reservoir concentrations compared to the transport time of each EV through the microchannels. We set our calculations under the following assumptions:

1. For the times less than zero, *t*< 0, there are no particles in either reservoir culture well (CW), peripheric channel (PC), or the microchannels (MC).
2. At time zero, *t*= 0, the concentrations of the conditioned media *C_CW_ = C_max_* (calculated by the raw DLS measurement numbers obtained), and *C_PC_ = 0*.
3. To apply the quasi-steady-state analysis, we assume that the time for culture media with EVs to diffuse across the microchannels (*t_MC_*) is much faster than the time for concentration to change in either wells (*t_W_*): *t_MC_*<<*t_W_*
4. Knowing that our smaller particle exomeres diffuse the fastest, to account for the most extreme scenarios, we based our calculations solely on this sub-type of EVs.
5. Second reservoir, the peripheric channel has an infinite volume (*V_PC_*= ∞).
6. The radius of the culture well *r_cw_*= 2 mm, height of the PDMS, *h_CW_*= 2 mm; where the shortest microchannel L_MC_ = 557 µm with height of *h_MC_* = 5 µm and width *w_MC_* = 20 µm.

Under these assumptions, the mathematical expression of the mass balance will simply be dictated by the flux (J) and the membrane area (A_m_) in our system shown in Eq. 2:

$-V_{cw}\frac{dC_{cw}}{\mathrm{dt}}=A_{MC}D_{particle}\left( \frac{C_{CW}-C_{PC}}{L_{MC}} \right)$ (Eq. 2)

Where the flux is (Eq.3):

$J=D_{particle}(\frac{C_{CW}-C_{PC}}{L_{MC}})$ (Eq.3)

In our experimental system, we conducted sample collection every 72 hours for the untreated neurons. In contrast, conditioned culture media from the OA-treated neurons were collected at 12-hour intervals to capture more rapid changes in EV transport. Solving for the percentage of the smallest particles (exomeres) transporting through the microchannels revealed that approximately 0.0005% of the total particle population diffused across the channel within 12 hours, increasing to approximately 0.003% over 72 hours.

**Small RNA-seq detailed methods and results**

The following tables and figures include details about the specific software pipeline used to perform differential analysis on the small RNA sequencing data to compare miRNA expression between healthy and treated neurons. Table S2 lists the software packages and databases and how they were used. Table S3 contains the alignment summary for each sample. Table S4 shows the size factors used to normalize the raw counts in the DESeq2 analysis, and Figure S10 shows the differences between the raw counts and the normalized counts for each sample. Table S5 and Table S6 contain details about the significantly dysregulated miRNAs as well as all the significantly expressed miRNAs for each cell type.

**Table S2: Software and databases used in the bioinformatics analysis.**

| Name | URL | Use |
| --- | --- | --- |
| CutAdapt | https://cutadapt.readthedocs.io/en/stable/guide.html | Adapter trimming and read filtering |
| FastQC | https://www.bioinformatics.babraham.ac.uk/projects/fastqc/ | Quality Control check of trimmed reads |
| Bowtie 1 | http://bowtie-bio.sourceforge.net/index.shtml | Read alignment |
| featureCounts | https://subread.sourceforge.net/ | Read counting |
| Samtools | http://www.htslib.org/ | Aligned file processing |
| DESeq2 | https://bioconductor.org/packages/release/bioc/html/DESeq2.html | Differential gene expression |
| iGenomes | https://support.illumina.com/sequencing/sequencing_software/igenome.html | Rnor_6.0 Ensembl reference genome; used for read alignment |
| miRbase | https://www.mirbase.org/download/ | Rnor genome coordinates (rno.gff3); used for read counting |

**Table S3: Bowtie Alignment Summary.**

| Sample | Reads Processed | Reads with at least one alignment | Reads that failed to align |
| --- | --- | --- | --- |
| C1-H | 956817 | 881978 (92.18%) | 74839 (7.82%) |
| C2-H | 976260 | 726412 (74.41%) | 249848 (25.59%) |
| C3-H | 464589 | 426701 (91.84%) | 37888 (8.16%) |
| C5-OA | 757561 | 681828 (90.00%) | 75733 (10.00%) |
| C7-OA | 970256 | 911058 (93.90%) | 59198 (6.10%) |
| C8-OA | 561290 | 505234 (90.01%) | 56056 (9.99%) |
| H1-H | 1001711 | 937708 (93.61%) | 64003 (6.39%) |
| H2-H | 1513746 | 1420519 (93.84%) | 93227 (6.16%) |
| H3-H | 1164658 | 1077092 (92.48%) | 87566 (7.52%) |
| H5 -OA | 561017 | 522698 (93.17%) | 38319 (6.83%) |
| H6-OA | 538183 | 506132 (94.04%) | 32051 (5.96%) |
| H7-OA | 708678 | 675184 (95.27%) | 33494 (4.73%) |
| S1-H | 845148 | 818202 (96.81%) | 26946 (3.19%) |
| S2-H | 1000682 | 965656 (96.50%) | 35026 (3.50%) |
| S4-H | 1325740 | 1293578 (97.57%) | 32162 (2.43%) |
| S5-OA | 678828 | 662341 (97.57%) | 16487 (2.43%) |
| S7-OA | 552529 | 534687 (96.77%) | 17842 (3.23%) |
| S8-OA | 689482 | 664021 (96.31%) | 25461 (3.69%) |

Note: After adapter trimming and size filtering, the reads for each sample were aligned to the Rnor_6.0 rat genome using Bowtie 1. Most of the reads aligned at least once to the genome. Samples are labelled with the cell type (Cortex = C, Hippocampus = H, Brainstem = S) and the treatment (Healthy = H, okadaic acid = OA).

**Table S4: DESeq2 size factor values for count normalization and differential expression analysis.**

| Sample | Size Factor |
| --- | --- |
| C1-H | 1.571 |
| C2-H | 1.338 |
| C3-H | 0.711 |
| C5-OA | 0.776 |
| C7-OA | 0.525 |
| C8-OA | 0.432 |
| H1-H | 1.818 |
| H2-H | 3.010 |
| H3-H | 2.046 |
| H5 -OA | 0.249 |
| H6-OA | 0.167 |
| H7-OA | 0.169 |
| S1-H | 2.097 |
| S2-H | 2.153 |
| S4-H | 3.811 |
| S5-OA | 1.867 |
| S7-OA | 1.426 |
| S8-OA | 1.601 |

Note: The size factors are calculated for each sample by the median of ratios method. The raw counts for each gene are then divided by the size factor. This normalization accounts for sequencing depth and RNA composition. Samples are label with the cell type (Cortex = C, Hippocampus = H, Brainstem = S) and the treatment (Healthy = H, okadaic acid = OA).

| 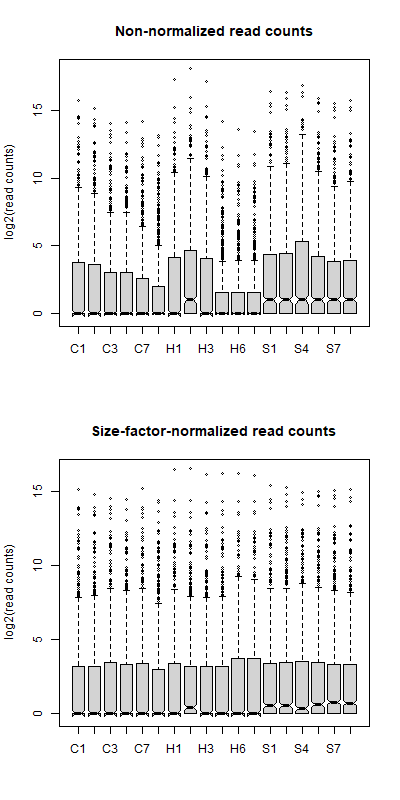 |
| --- |
| Figure S10. Distribution of raw counts (top) compared to the normalized counts (bottom) after dividing by the size factors in Table S4. The normalized counts have a more similar distribution across samples, which is the expected result. |

**Table S5: Brain tissue region sorted miRNAs based on significant expressions**

| **Cortex, Hippocampus, and Brainstem** |
| --- |
| let-7a-1-3p, let-7a-5p, let-7b-3p, let-7b-5p, let-7c-1-3p, let-7c-2-3p, let-7c-5p, let-7d-3p, let-7e-3p, let-7f-5p, let-7g-5p, let-7i-5p    miR-1b, miR-7a-5p, miR-20a-5p, miR-21-5p, miR-22-3p, miR-22-5p, miR-23a-3p, miR-23b-3p, miR-24-2-5p, miR-24-3p, miR-25-3p, miR-26a-5p, miR-26b-5p, miR-27a-3p, miR-27a-5p, miR-27b-3p, miR-27b-5p, miR-28-3p, miR-30a-3p, miR-30a-5p, miR-30c-2-3p, miR-30c-5p, miR-30d-5p, miR-30e-3p, miR-30e-5p, miR-34c-5p, miR-92a-3p, miR-92b-3p, miR-92b-5p, miR-93-5p, miR-98-5p, miR-99a-5p, miR-99b-3p, miR-99b-5p,    miR-100-5p, miR-101b-3p, miR-103-3p, miR-106b-3p, miR-124-3p, miR-125a-3p, miR-125a-5p, miR-125b-1-3p, miR-125b-2-3p, miR-125b-5p, miR-126a-3p, miR-127-3p, miR-127-5p, miR-128-3p, miR-129-1-3p, miR-129-5p, miR-1298, miR-130b-5p, miR-132-3p, miR-132-5p, miR-134-5p, miR-135b-5p, miR-136-5p, miR-139-5p, miR-140-3p, miR-140-5p, miR-146b-5p, miR-148a-3p, miR-148b-3p, miR-149-5p, miR-152-3p, miR-155-5p, miR-16-5p, miR-17-5p, miR-181a-2-3p, miR-181a-5p, miR-181b-5p, miR-181d-5p, miR-185-5p, miR-186-5p, miR-187-3p, miR-191a-5p, miR-192-5p, miR-194-5p, miR-195-3p    miR-204-3p, miR-204-5p, miR-211-5p, miR-212-5p, miR-218a-5p, miR-219a-1-3p, miR-221-3p, miR-221-5p, miR-222-3p, miR-296-3p, miR-298-3p, miR-298-5p, miR-300-3p, miR-301a-5p, miR-320-3p, miR-322-3p, miR-328a-3p, miR-328b-3p, miR-329-3p, miR-329-5p, miR-330-3p, miR-330-5p, miR-339-3p, miR-339-5p, miR-340-3p, miR-340-5p, miR-341, miR-342-3p, miR-342-5p, miR-344a-3p, miR-344b-1-3p, miR-345-3p, miR-346, miR-351-5p, miR-3559-3p, miR-361-3p, miR-361-5p, miR-363-3p, miR-369-3p, miR-370-3p, miR-374-5p, miR-379-3p, miR-379-5p, miR-381-3p, miR-382-5p, miR-383-5p, miR-384-5p    miR-409a-5p, miR-410-3p, miR-411-3p, miR-411-5p, miR-421-3p, miR-423-3p, miR-423-5p, miR-425-5p, miR-431, miR-433-3p, miR-434-5p, miR-455-3p, miR-455-5p, miR-484, miR-485-3p, miR-485-5p, miR-487b-3p, miR-488-3p, miR-493-3p, miR-493-5p, miR-494-3p, miR-495, miR-499-5p, miR-503-3p, miR-503-5p, miR-505-5pmiR-532-5p, miR-540-3p, miR-541-5p, miR-542-3p, miR-543-3p, miR-598-3p, miR-652-3p, miR-671, miR-672-5p, miR-674-3p, miR-674-5p, miR-676, miR-708-3p, miR-708-5p, miR-760-3p, miR-770-3p, miR-872-5p, miR-1839-5p, miR-1843b-5p, miR-6331 |
| **Cortex and Hippocampus** |
| miR-124-5p, miR-128-2-5p, miR-136-3p, miR-137-3p, miR-137-5p, miR-138-1-3p, miR-138-2-3p, miR-138-5p, miR-139-3p, miR-181c-5p, miR-212-3p, miR-296-5p, miR-324-3p, miR-323-3p, miR-331-3p, miR-380-3p, miR-412-5p, miR-376b-3p, miR-664-2-5p, miR-664-3p, miR-667-3p, miR-668, miR-758-3p, miR-770-5p, miR-1188-5p, miR-6321 |
| **Cortex and Brainstem** |
| miR-135a-3p, miR-146a-5p, miR-206-3p, miR-3068-5p, miR-323-5p, miR-375-3p, miR-449a-5p, miR-874-3p, miR-877, miR-935 |
| **Hippocampus and Brainstem** |
| miR-1843b-3p, miR-195-5p, miR-323-5p, miR-374-3p, miR-450b-5p |
| **Cortex** |
| miR-7b, miR-130b-3p, miR-199a-5p, miR-216b-5p, miR-344b-5p, miR-490-3p, miR-666-5p, miR-667-5p, miR-1306-3p, miR-3099 |
| **Hippocampus** |
| miR-19b-3p, miR-29c-5p, miR-135b-3p, miR-153-3p, miR-182, miR-324-5p, miR-326-3p, miR-362-5p, miR-376a-5p, miR-1843a-5p, miR-3099 |
| **Brainstem** |
| let-7f-1-3p, miR-15b-3p, miR-15b-5p,miR-23b-5p, miR-28-5p, miR-29b-3p, miR-32-5p, miR-34a-5p, miR-34b-3p, miR-34b-5p, miR-142-3p, miR-142-5p, miR-143-3p, miR-143-5p, miR-145-3p, miR-145-5p, miR-150-5p, miR-184, miR-199a-3p, miR-199a-5p, miR-200c-3p, miR-205, miR-214-3p, miR-223-5p, miR-224-5p, miR-365-3p, miR-365-5p, miR-449c-5p, miR-450b-3p, miR-872-3p, miR-3559-5p |

Note: Significant expression is defined here as having at least five normalized counts in three or more samples for each cell type. Most of the significantly expressed miRNAs are present in all three cell types. Then there are 31 miRNAs significantly expressed only in the brainstem and 26 miRNAs significantly expressed in only the cortex and hippocampus.

**Table S6: List of significantly dysregulated miRNAs based on Log2 fold change, the log fold change standard error, average expression, and adjusted p-value.**

| miRNA | Cell Type | Log2 FoldChange | lfcSE | Average Expression | Adjusted p-value |
| --- | --- | --- | --- | --- | --- |
| miR-21-5p | Cortex | 1.450 | 0.276 | 12049.49 | 4.69E-06 |
|  | Hippocampus | 2.394 | 0.276 | 12049.49 | 5.66E-16 |
| miR-22-3p | Cortex | 0.794 | 0.255 | 331.524 | 3.15E-2 |
| miR-23a-3p | Cortex | 2.159 | 0.388 | 114.661 | 1.19E-06 |
|  | Hippocampus | 2.261 | 0.398 | 114.661 | 4.34E-07 |
| miR-24-3p | Hippocampus | 1.006 | 0.198 | 1465.118 | 1.10E-05 |
| miR-27a-3p | Cortex | 1.817 | 0.376 | 263.899 | 3.39E-05 |
|  | Hippocampus | 2.498 | 0.366 | 263.8989 | 4.32E-10 |
| miR-27a-5p | Cortex | 2.401 | 0.455 | 44.0991 | 4.69E-06 |
|  | Hippocampus | 3.339 | 0.584 | 44.0991 | 4.12E-07 |
| miR-128-2-5p | Cortex | -1.595 | 0.442 | 14.618 | 5.93E-3 |
| miR-129-1-3p | Cortex | 1.458 | 0.314 | 26.413 | 7.37E-05 |
|  | Hippocampus | 1.208 | 0.385 | 26.413 | 3.42E-2 |
| miR-129-5p | Cortex | 1.031 | 0.204 | 2072.864 | 1.19E-05 |
|  | Hippocampus | 1.514 | 0.206 | 2072.864 | 1.13E-11 |
| miR-132-3p | Cortex | 1.809 | 0.208 | 233.999 | 7.13E-16 |
|  | Hippocampus | 2.067 | 0.211 | 233.999 | 2.84E-20 |
| miR-132-5p | Cortex | 1.735 | 0.256 | 98.623 | 1.19E-09 |
|  | Hippocampus | 1.943 | 0.261 | 98.623 | 8.15E-12 |
|  | Brainstem | 1.225 | 0.300 | 98.623 | 1.16E-2 |
| miR-146b-5p | Brainstem | 0.969 | 0.147 | 441.532 | 2.01E-08 |
| miR-155-5p | Cortex | 4.318 | 0.694 | 56.498 | 2.83E-08 |
|  | Hippocampus | 5.716 | 0.723 | 56.498 | 2.69E-13 |
| miR-212-3p | Cortex | 3.314 | 0.688 | 9.757 | 3.39E-05 |
|  | Hippocampus | 3.281 | 0.648 | 9.757 | 1.12E-05 |
| miR-212-5p | Cortex | 1.903 | 0.282 | 70.774 | 1.19E-09 |
|  | Hippocampus | 1.934 | 0.299 | 70.774 | 4.35E-09 |
| miR-342-3p | Hippocampus | 0.885 | 0.249 | 117.789 | 8.78E-03 |
| miR-351-5p | Brains tem | -0.818 | 0.216 | 270.038 | 2.58E-02 |
| miR-362-5p | Hippocampus | 2.406 | 0.702 | 6.211 | 1.30E-02 |
| miR-431 | Cortex | 1.351 | 0.435 | 14.683 | 3.15E-02 |
| miR-664-2-5p | Hippocampus | 2.434 | 0.669 | 7.848 | 6.89E-03 |

Note: DESeq2 finds an initial p-value using the Wald Test, then corrects this p-value for the multiple testing problem (where the more genes tested lead to a higher false positive rate) by the Benjamini and Hochberg method.

References:

1. A. Magnaudeix, C. M. Wilson, G. Page, C. Bauvy, P. Codogno, P. Lévêque, F. Labrousse, M. Corre-Delage, C. Yardin and F. Terro, *Neurobiology of Aging*, 2013, **34**.

2. A. Kunze, R. Meissner, S. Brando and P. Renaud, *Journal*, 2011, **108**.

3. Z. Zhang and J. W. Simpkins, *Brain Research*, 2010, **1345**.

4. S. Y. Yoon, J. E. Choi, H. S. Kweon, H. Choe, S. W. Kim, O. Hwang, H. Lee, J. Y. Lee and D. H. Kim, *Journal of Neuroscience Research*, 2008, **86**.

5. I. Holen, P. B. Gordon and P. O. Seglen, *Biochemical Journal*, 1992, **284**.

6. X. Wang, Y. Zhou, Q. Gao, D. Ping, Y. Wang, W. Wu, X. Lin, Y. Fang, J. Zhang and A. Shao, *Journal*, 2020, **2020**.

7. M. Gatti, M. Zavatti, F. Beretti, D. Giuliani, E. Vandini, A. Ottani, E. Bertucci and T. Maraldi, *Oxidative Medicine and Cellular Longevity*, 2020, **2020**.
